# A magnetic antibody-conjugated nano-system for selective delivery of Ca(OH)_2_ and taxotere in ovarian cancer cells

**DOI:** 10.1038/s42003-022-03966-w

**Published:** 2022-09-21

**Authors:** Reza Taheri-Ledari, Ehsan Zolfaghari, Simindokht Zarei-Shokat, Amir Kashtiaray, Ali Maleki

**Affiliations:** Catalysts and Organic Synthesis Research Laboratory, Department of Chemistry, Iran University of Science and Technology, Tehran, 16846-13114 Iran

**Keywords:** Drug delivery, Targeted therapies

## Abstract

An efficient strategy for cancer therapy is presented, in which a tumor mass is initially pretreated with calcium hydroxide, then treated with Taxotere (TXT). In this regard, an advanced delivery system based on iron oxide nanoparticles has been designed. The surface of nanoparticles was functionalized with sortilin (SORT-1, a human IgG1 monoclonal antibody) that specifically encodes caov-4 ovarian cancerous cells. Plasmonic heating of the incorporated gold nanoparticles in polyvinyl alcohol (PVA) has been exploited to control the release process of TXT. The in vitro, ex vivo and in vivo experiments have exhibited high efficacy of a seven-day pretreatment by Ca(OH)_2_ plus 14 days treatment program by Ca(OH)_2_@Fe_3_O_4_/PVA/Au-SORT nano-therapeutics, where more penetration ratio resulted in tumor growth inhibition by ca. 78.3%. As a result, due to showing high values of the anti-tumor properties and biosafety, the presented pretreatment strategy is suggested for more effective treatment on the aged tumors.

## Abstract (web summary)

A magnetic drug delivery system containing polyvinyl alcohol, gold nanoparticles, and sortilin antibody followed by the plasmonic photothermal heating strategy for the controlled drug release is proposed, with use in ovarian cancer demonstrated.

## Introduction

Today, traditional chemotherapy of the diverse types of cancers is gradually being converted to a safer version of methodology by enhancing the targeted drug delivery to the cancerous tumors^1–3^. Targeted drug delivery has been experimentally achieved by the executing different efficient strategies that were someday in the scientists’ dreams. As an instance of targeting methods, folate-mediated drug delivery is a highly selective method for targeted treatment of cancer diseases due to overexpression of the folate receptor by ovarian carcinomas^4^. For colon targeted drug delivery, Chourasia and Jain.^5^ published a report in which interesting methods such as covalent linkage of a drug with a carrier degradable by colonic bacteria, coating with pH-responsive polymers, and special formulation methods affecting the release time, have been discussed As another example, protein-drug conjugates^6^ [in particular, antibody-drug conjugates (ADCs)], a well-known generation of high-tech pharmaceutical compounds include targeting function through antibodies in their structures^7,8^. As a brief explanation, antibodies show an exclusive attachment to their specific receptors (antigens) located onto the cell membrane (following the key-lock pattern), resulting in a great targeting in drug delivery applications, especially in cancer therapy^9^. In ADCs, the drug is directly connected to a specific antibody by an organic structure (as linker), which may be a protein/peptide strand, aliphatic hydrocarbon, and a polymeric chain^10,11^. Although this strategy has exhibited substantial targeting in drug delivery and seemed to be the most efficient method ever, there may be some drawbacks. For example, direct contact of the ADCs with the free glutamates present in the blood serum may result in de-conjugation and subsequently immediate release of the cytotoxic drug^12^. In contrast, in the nanoscale cargoes, the carried drug is well wrapped and would not be released if de-conjugation of the antibody (located onto the surfaces) is occurred. Moreover, in the case of the ADCs, only using a sensitive linker between drug and the antibody can be considered for drug release and there would be no so strong control over the drug release process^13^, while a strong controlling using plasmonic gold nanoparticles (AuNPs) can be achieved in the nanoscale cargoes^14,15^. As one of the most important matters, tumor penetration is almost a physical capability, which the metallic nanoparticles possess instead of the ADCs^16^. Another excellence that exclusively belongs to the magnetic drug carriers is a physical direction and further accumulation in tumor tissue and the biological aggregation, which is considered as synergy in targeted drug delivery^17,18^. As another case, the lifetime of the carried drug is optimized with fewer limitations in the nanoscale cargoes than in the ADCs because in the ADCs, only the length of the linker can be manipulated^19^, while in the nanoscale cargoes we would have more options to do that (e.g., using different polymeric matrices)^20^. All of the mentioned concerns led researchers to modify the ADCs strategy via a combination of biotechnology with nanotechnology. As a result, so many efficient drug delivery systems have been reported in the recent decade. The surface of different types of the nanoscale materials such as metal-organic frameworks^21,22^, carbon^23,24^ halloysite nanotubes^25,26^, and metallic nanoparticles^27–29^ have been functionalized with antibodies to add targeting function to their systems.

Recently, we have studied the synergistic therapeutic effects of applying magnetic direction of the cargos to the targeted tissue and controlled release of Taxotere (TXT) by plasmonic photothermal stimulation of the AuNPs^30^. The AuNPs play a dual-edged role in the cancer therapy: (1) tumor metastasis inhibition that is considered as a biological function^31^, and (2) plasmonic photothermal therapy (PPTT) that is implemented through an exclusive capability of the AuNPs calling LSPR effect (LSPR stands for localized surface plasmonic resonance), and is considered as a physicochemical property of the AuNPs^32,33^. PPTT provides a fantastic opportunity to efficiently penetrate the adipose tissue of the cancerous masses by heating the AuNPs under irradiation of a specific wavelength^34^. This method converts the AuNPs to hot spots that could trigger a drug release program in the composites with high controlling, as well^35^. Moreover, there are other benefits to the use of AuNPs in the structure of the therapeutic composites, which contain a cytotoxic drug. For example, detection of the drug cargo in the body’s internal environment becomes feasible by computed tomography imaging due to the high contrast of the AuNPs in the X-ray scan images^36,37^.

According to literature, calcium hydroxide can effectively alter the properties of the lipid-base biological structures due to its alkaline nature^38^. Besides, partial dosages of this mineral compound are quite safe for healthy organs^39^. As an instance of the important role of calcium hydroxide in dental sciences, breaking the bacterial complex lipids that have been reported to be inflammatory activators can be referred^40^. The main use indications of calcium hydroxide in cancer therapy is the dissolution of necrotic tissue, which are mostly correlated to its high pH and necrotizing capacity^41,42^. According to literature, destruction of epithelium present in periradicular lesions by calcium hydroxide leads to the enhanced penetrability of the aged tumors^43^. In fact, calcium hydroxide can reduce the hydrophobicity of tumors via the destruction of the fatty pads, through its alkaline nature^44^. Hence, we decided to test the potential effects of this mineral on the dissociation of the massed tumor to make it softer and more penetrable for further treatment by TXT.

Based on the descriptions above, herein, we suggest an applicable method for pretreatment of the aged cancerous tumors to improve its properties and prepare that for better drug reception. For this purpose, a low dosage of calcium hydroxide (Ca(OH)_2_) is administrated to the tumor-bear mice by applying a developed drug delivery strategy. Then, the main treatment is performed via selective delivery and controlled release of TXT as a cytotoxic drug. For selective delivery of Ca(OH)_2_ and TXT to the cancer cells (caov-4 cells), a new therapeutic composite constructed of iron oxide nanoparticles (Fe_3_O_4_ NPs) as a biodegradable magnetic core, polyvinyl alcohol (PVA) as a proper matrix for encapsulation of the drugs, AuNPs for PPTT application and controlled release of the encapsulated TXT, and human IgG1 sortilin 2D8-E3 monoclonal antibody (SORT), has been used. Previously, we have demonstrated high efficiency of the Fe_3_O_4_/PVA core/shell structure for drug delivery applications^45^. This work intends to precisely investigate the possible therapeutic synergies by selective targeting, magnetic direction, and PPTT release of the loaded TXT and apoptosis effects. For this aim, TXT@Fe_3_O_4_/PVA/Au-SORT nano-therapeutic has been prepared in cold conditions, characterized by various analyses, and utilized for cancer growth inhibition. Briefly, in vitro, ex vivo, and in vivo experiments have revealed well growth inhibition potency for TXT@Fe_3_O_4_/PVA/Au-SORT nano-therapeutic. Also, confocal microscopy has verified high selectivity in cellular uptake and internalization processes. Moreover, high anti-tumor properties and biosafety of the prepared therapeutic cargoes have been corroborated via execution of a 7-day pretreatment with the low dosages of Ca(OH)_2_@Fe_3_O_4_/PVA/Au-SORT, and further a 14-day treatment by TXT@Fe_3_O_4_/PVA/Au-SORT nano-therapeutic. Bioluminescence and fluorescence imaging on the excised tumors and living mice have confirmed the high efficacy of the presented method.

## Results and discussion

### Preparation of TXT@Fe_3_O_4_/PVA/Au-SORT nano-therapeutics

**Fig. 1:**
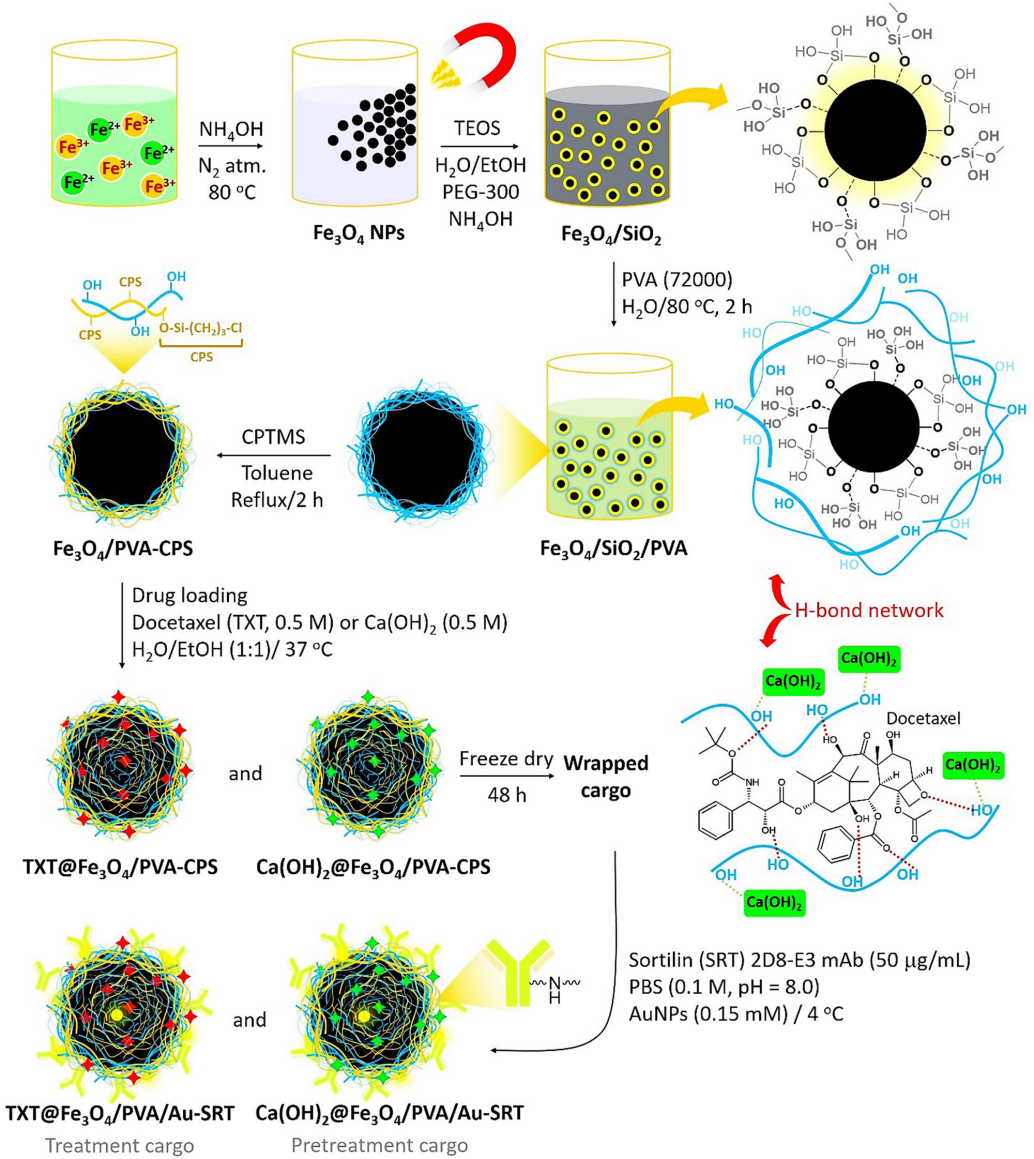
Schematic presentation of preparation route of TXT@Fe_3_O_4_/PVA/Au-SORT nano-therapeutics. TEOS stands for tetraethylorthosilicate; PVA stands for polyvinyl alcohol; CPTMS stands for 3-chloropropyl trimethoxysilane; CPS stands for chloropropylsilane; red stars show TXT drug; green stars show calcium hydroxide; yellow circles show AuNPs; covalent bonding is occurred between the C-Cl bonds onto the surfaces (CPS) and amine groups in the structure of the antibody, lysine amino acids.

The preparation route of the TXT@Fe_3_O_4_/PVA/Au-SORT nano-therapeutic has been schematically presented in Fig. 1. As shown, the magnetic particles of the Fe_3_O_4_ were obtained via a co-deposition method in alkaline conditions^46,47^. Next, the particles were coated by a silica (SiO_2_) network to prevent the oxidation of the particles to Fe_2_O_3_, which is less magnetic than Fe_3_O_4_, and also to increase the stability against intense conditions^48–50^. Afterward, composition with the PVA strands was performed in the aqueous medium. The main contributor to this composition is tight H-binding interactions between the hydroxyl (-OH) groups, which are abundantly present in the PVA structure and on the Fe_3_O_4_@SiO_2_ surfaces^51^. In the next stage, the surfaces of the Fe_3_O_4_@SiO_2_-PVA particles were modified by (3-chloropropyl) trimethoxy silane (CPTMS). In this stage, the exterior -OH groups onto the surfaces attack the silane element of CPTMS, and the methoxy groups leave the structure. Generally, two goals are pursued with the modification by the CPTMS: (1) providing an appropriate available chemical site (C–Cl) for the covalent attachment of the antibody, and (2) increasing the hydrophobicity of the surface through the addition of -(CH_2_)_3_- groups, which is highly preferred in drug delivery to the cancer tumors^52^. The next step is the encapsulation of calcium hydroxide (as pretreatment cargo) and TXT (as treatment cargo) into the PVA matrix. The PVA network swells by the hydration process at higher temperatures, and a large amount of the TXT could be incorporated into this jelly matrix^53^. At this stage, the TXT molecules that include several active functional groups such as hydroxyl, ester, and amide (including oxygen and nitrogen heteroatoms) are immobilized in the PVA network through strong H-bond interactions with the hydroxyl groups present in PVA structure^54^. In the case of calcium hydroxide, tight electrostatic interactions between the calcium and oxygen atoms hold calcium hydroxide in the PVA network^55^. Furthermore, jelly nature of the PVA layer enhances the adsorption of the Ca(OH)_2_ and TXT into the network^56^. As presented in Fig. 1, Ca(OH)_2_@Fe_3_O_4_@PVA-CPS and TXT@Fe_3_O_4_@PVA-CPS cargoes are well wrapped by freeze dryer (for 48 hours), through which the PVA layer is completely shrunk at a very low temperature. Hence, it can be expressed that both physical and chemical factors play the key roles in complexation of the Ca(OH)_2_ and TXT into the PVA layer. After this step, the drug cargo was well wrapped by freeze drier at the low temperatures, and finally were redispersed in the PBS buffer for the conjugation of the SORT antibody and also incorporation of the as-prepared AuNPs into the PVA matrix. For the conjugation of the SORT antibody to the prepared TXT@Fe_3_O_4_@PVA-CPS nanocomposite, the present amine groups were activated in the alkaline condition and attack the C–Cl sites onto the surfaces.

### Structural investigations and characterization of TXT@Fe_3_O_4_/PVA/Au-SORT nano-therapeutics

At the first stage, to evaluate surface modification of the magnetic particles, and TXT-loading into the PVA network, Fe_3_O_4_@SiO_2_, Fe_3_O_4_@SiO_2_-PVA, and TXT@Fe_3_O_4_/PVA/Au-SORT samples were studied by Fourier-transform infrared (FTIR), energy-dispersive X-ray (EDX), photoluminescence (PL) emission and ultraviolet-visible differential reflectance spectroscopy (UV-DRS). All obtained results from these analyses have been precisely interpreted and fully presented in Supporting Information (SI) section (Figs. S1–3). Also, magnetic properties of the TXT@Fe_3_O_4_/PVA/Au-SORT nanoparticles (in comparison with the controls) were studied by vibrating-sample magnetometer (VSM), and the obtained results have been carefully presented in the SI section (Figure S4). Calculations of the ratios of different components in the presented drug delivery system based on CHN and EDX analyses, and drug content have been presented in the SI section (Supplementary Note 1).

**Fig. 2:**
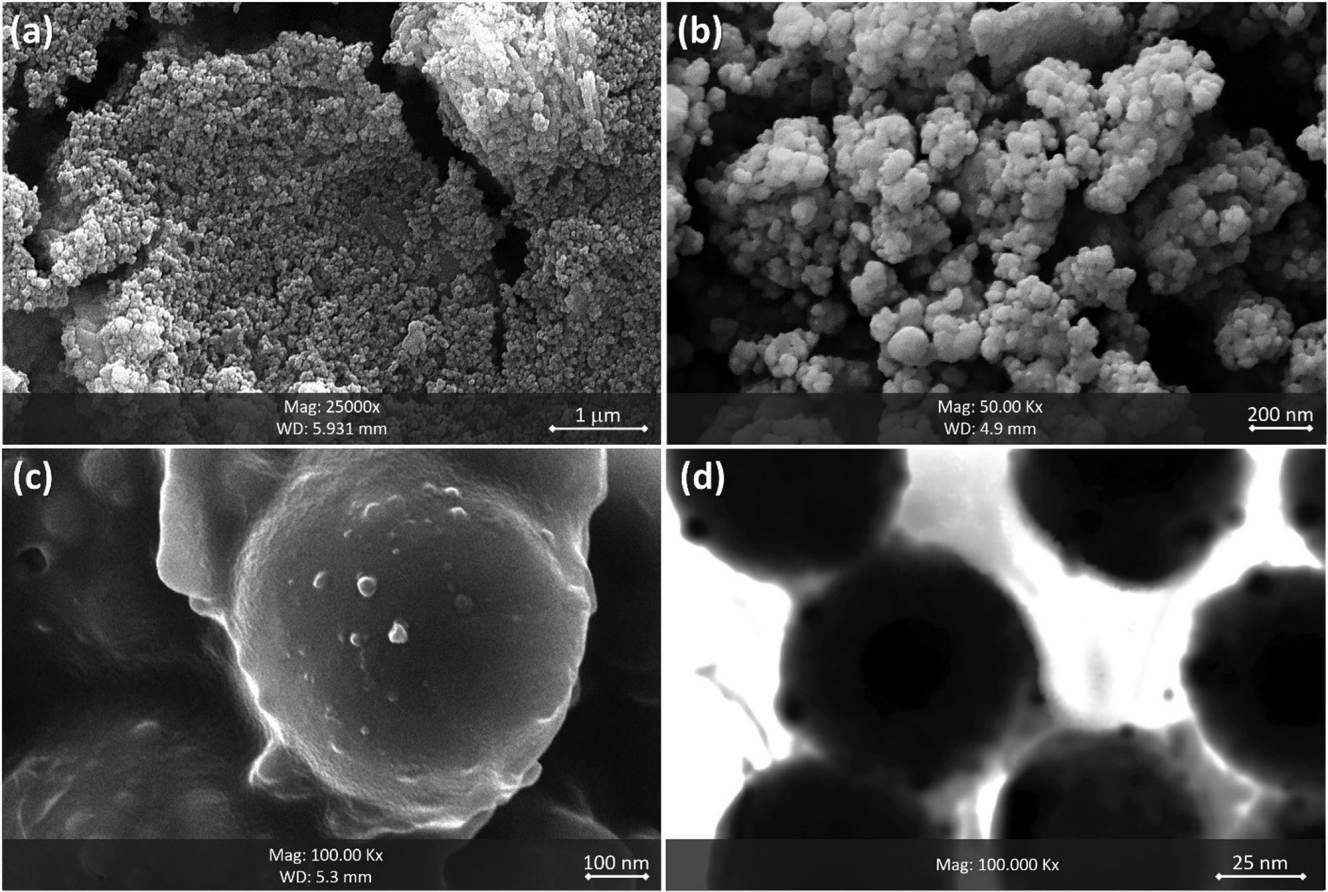
FESEM and TEM images of the prepared products within the successive stages. **a** SEM image of Fe_3_O_4_ NPs, **b** Fe_3_O_4_@SiO_2_ NPs, and **c** TXT@Fe_3_O_4_/PVA/Au-SORT nanocargo. **d** The TEM image of TXT@Fe_3_O_4_/PVA/Au-SORT nanocargo.

To investigate the size and morphology of the particles, scanning-electron microscopy (SEM) and field-emission scanning-electron microscopy (FESEM) instruments were used. As illustrated in Fig. 2a, b, well dispersion of the particles of Fe_3_O_4_@SiO_2_ with high uniformity in size and shape has been obtained via ultrasonication by a cleaner bath (50 KHz, 200 W L^−1^). The mean size of these particles with a spherical morphology was estimated in ca. 38 nm. As expected, the composition of the particles with the PVA strands led to the aggregation of the particles of Fe_3_O_4_@SiO_2_-PVA (Fig. 2c). In fact, a synergy has occurred between the magnetic property of the particles and the inherent jelly nature of the PVA matrix for the particles aggregation. The formed masses of the particles of Fe_3_O_4_@SiO_2_-PVA, which have been surrounded by the PVA matrix, are clearly observed in panel (c). To remove the excess PVA from the Fe_3_O_4_@SiO_2_-PVA composite and prepare that for further modification by CPTMS, the particles of the Fe_3_O_4_@SiO_2_-PVA were well washed with the tepid deionized water (~30 °C) several successive times, dried by freeze dryer, and then the FESEM imaging was performed on the sample. The mean size of PVA-coated particles was estimated in ca. 550 nm, showing a significant increase in the size of the particles after coating by the PVA. Figure 2c illustrates the FESEM image of TXT@Fe_3_O_4_/PVA/Au-SORT particles including brilliant spots on the spheres (into the PVA network), which verify the presence of the AuNPs in the structure. The incorporated AuNPs in the structure is also seen in the TEM image of TXT@Fe_3_O_4_/PVA/Au-SORT (Fig. 2d) as the dark spots. From the TEM image, the average size of the AuNPs is estimated to be ca. 8–15 nm, which is in a well correspondence with the TEM image of the as-prepared individual AuNPs (Figure S5, in the SI file). Moreover, the red color of the colloidal solution of the AuNPs confirms that the mean size of the particles is so close to 10–15 nm (Figure S6, in the SI file). To investigate the stability of the particles and also dispersion/aggregation states in the colloidal phase, dynamic-light scattering (DLS) analysis was performed on the diluted samples of TXT@Fe_3_O_4_/PVA/Au-SORT. For this purpose, the buffered samples (PBS, 0.1 M, pH = 7.3) of TXT@Fe_3_O_4_/PVA/Au-SORT with concentrations of 10, 20, 30, and 50 μg/mL were ultrasonicated by a probe sonicator (50 KHz and 150 W L^−1^), and then studied by zeta-sizer. Based on the obtained results from DLS measurements (presented in the SI section, Figure S7), the most suitable dispersion state was observed in the case of 50 μg/mL concentration.

### Therapeutic features of TXT@Fe_3_O_4_/PVA/Au-SORT nano-therapeutic

#### Screening TXT content and of PPTT-triggered release from TXT@Fe_3_O_4_/PVA/Au-SORT system

To investigate how much TXT is incorporated in the composite, UV-vis spectroscopy was used. For this purpose, a calibration curve was initially obtained from UV-vis absorption activity of five standard solutions of TXT in PBS (0.1, pH = 7.3) with the concentrations of 5, 10, 15, 25, and 30 ppm, at *λ*_max_ = 230 nm (Figure S8, in the SI section)^57^. According to the calculations give in the SI section, drug content of the TXT@Fe_3_O_4_/PVA/Au-SORT system was estimated to be ca. 13.3 wt%.

**Fig. 3:**
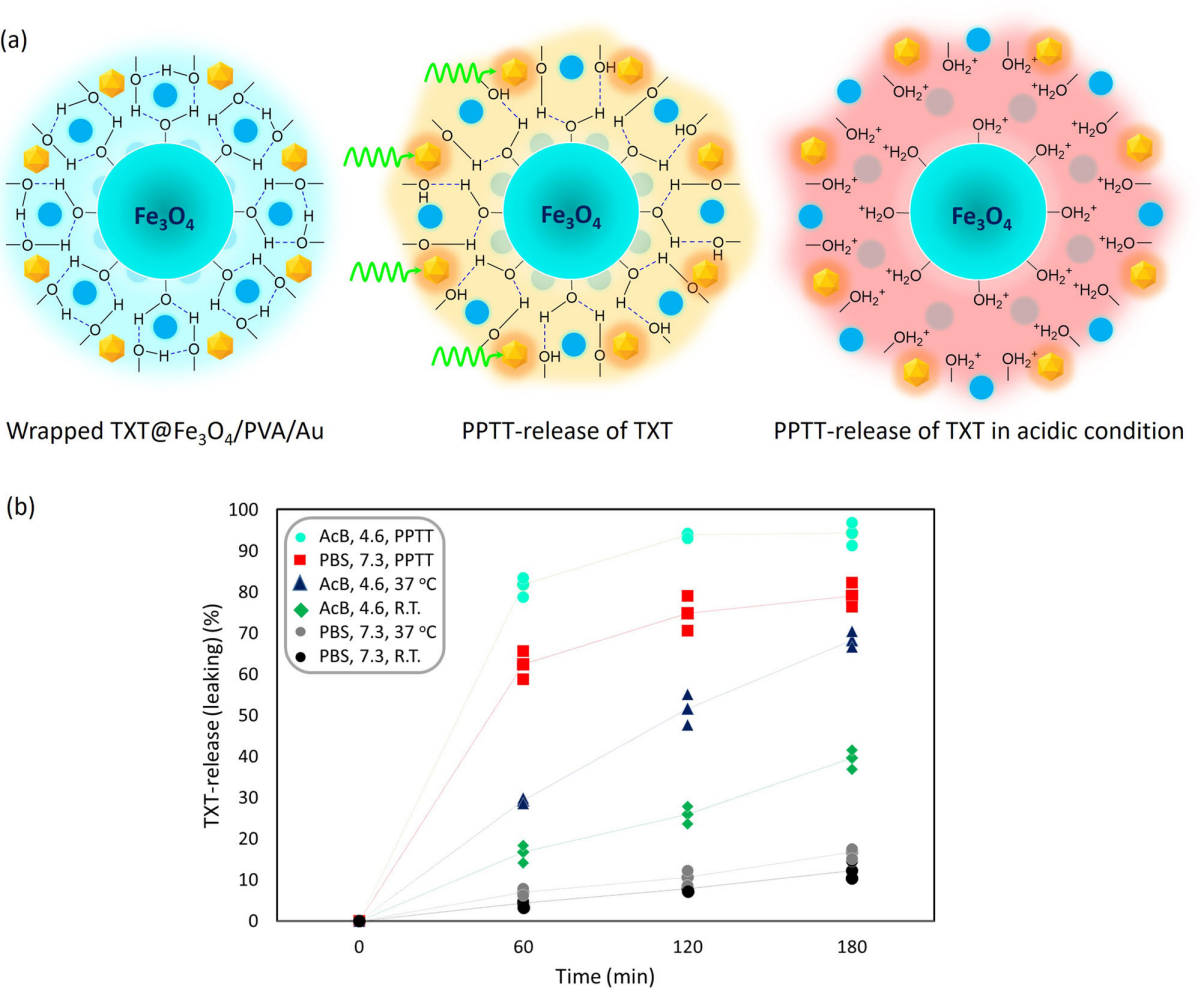
Schematic presentation of dissociation of the PVA matrix and subsequent drug release. **a** Dissociation of the cross-linkages of the PVA substrate at high temperatures (provided by AuNPs exposed to green LED light) and acidic conditions (provided by AcB, pH = 4.6). **b** TXT-release (or leaching) profile at different conditions during a 180-minute process.

To investigate the release states (or leaching) of the loaded TXT from the Fe_3_O_4_/PVA/Au-SORT drug carrier system, a simulated circulatory system formed of a circulatory pump, a cylindrical glass container, a green LED light source (7 W, *λ* = 526 nm), and the transparent silicone vessels, was used^58^. For this purpose, the TXT@Fe_3_O_4_/PVA/Au-SORT particles were dispersed in related buffer media via ultrasonication, and the specific conditions were applied to the cylindrical glass container. The influence of the different factors such as medium, time, and conditions on the drug release process was carefully monitored by magnetic separation of the particles after completion of the process, filtration, and UV-vis spectroscopy at 235 nm (*λ*_max_ of TXT)^57,59^. Finally, the obtained data from the UV-vis spectroscopy were analyzed by using the line equation of the calibration curve, which was presented in the previous section. Table 1 summarizes the obtained values for the TXT-release/leaching in the designed circulatory system under different conditions. As is seen in the table, the highest value of TXT-release (94.4 ± 4.1) % has been obtained in an acidic medium (AcB, pH = 4.6) during 180 minutes, under PPTT conditions provided by the green LED light (entry 14). As is seen, (81.8 ± 5.1) % release value has occurred in 60 minutes, which corroborates high control in the release process (entry 12). Also, it is confirmed that partial amounts of the loaded TXT are leached from the carrier in the neutral pH and 37 °C, which is a similar environment to the blood serum (entries 2–4). Moreover, the responsibility of the PVA matrix to high temperatures (PPTT heating) and acidic media (AcB) have been separately demonstrated in the table (entries 5–11). Figure 3a schematically presents the PVA dissociation process and subsequent release of TXT incorporated into the PVA matrix via PPTT heating in an acidic medium. Also, Fig. 3b exhibits the release profile of the TXT during a 180-minute process under different conditions. As can be observed at the dot-curves, PPTT heating by a green LED light has led to immediate release, as the major amount of the loaded TXT in the system has been released during 60 minutes. In addition to the PPTT conditions, this is observed that the acidic condition of AcB has led to an almost complete release than the PBS. To investigate the stability of the Fe_3_O_4_/PVA/Au composite particles as the main skeleton of the designed TXT@Fe_3_O_4_/PVA/Au-SORT nano-cargo, different conditions and media were experimented in a simulated circulatory system including a bilateral pump, cylindrical glass container, and silicon vessels. All details and the obtained results have been given in the SI section (Figure S9 and Table S3).

**Table 1:** Screening of TXT release/leaching values from TXT@Fe_3_O_4_/PVA/Au-SORT system in a simulated circulatory system, under different conditions.

Entry	Medium	pH	Conditions	Time (min)	Released/leached TXT (±error) (%)
1	PBS	7.3	Room temperature	180	12.2 ± 1.5
2	PBS	7.3	37 °C	60	7.0 ± 1.6
3	PBS	7.3	37 °C	120	10.6 ± 1.3
4	PBS	7.3	37 °C	180	16.8 ± 1.2
5	PBS	7.3	Green LED light irradiation	60	62.4 ± 3.1
6	PBS	7.3	Green LED light irradiation	120	74.8 ± 3.6
7	PBS	7.3	Green LED light irradiation	180	79.1 ± 4.8
8	AcB	4.6	Room temperature	180	39.7 ± 1.7
9	AcB	4.6	37 °C	60	29.3 ± 2.3
10	AcB	4.6	37 °C	120	51.6 ± 2.8
11	AcB	4.6	37 °C	180	68.2 ± 3.7
12	AcB	4.6	Green LED light irradiation	60	81.8 ± 5.1
13	AcB	4.6	Green LED light irradiation	120	94.0 ± 6.2
14	AcB	4.6	Green LED light irradiation	180	94.4 ± 8.5^*^

^*^Optimum condition; ultrasound bath (50 KHz, 100 W L^−1^); PBS and AcB concentration is 0.1 M; relative errors belong to three samples for each condition; PPTT; irradiation was performed only for a half of the total time (10 minutes irradiation in each 20 minutes circulation), a green LED light 7.0 W with the specific wavelength of 526 nm was used. The cylindrical glass container was irradiated.

#### Cellular investigation of selective uptake of TXT@Fe_3_O_4_/PVA/Au-SORT nano-therapeutic by confocal microscopy

**Fig. 4:**
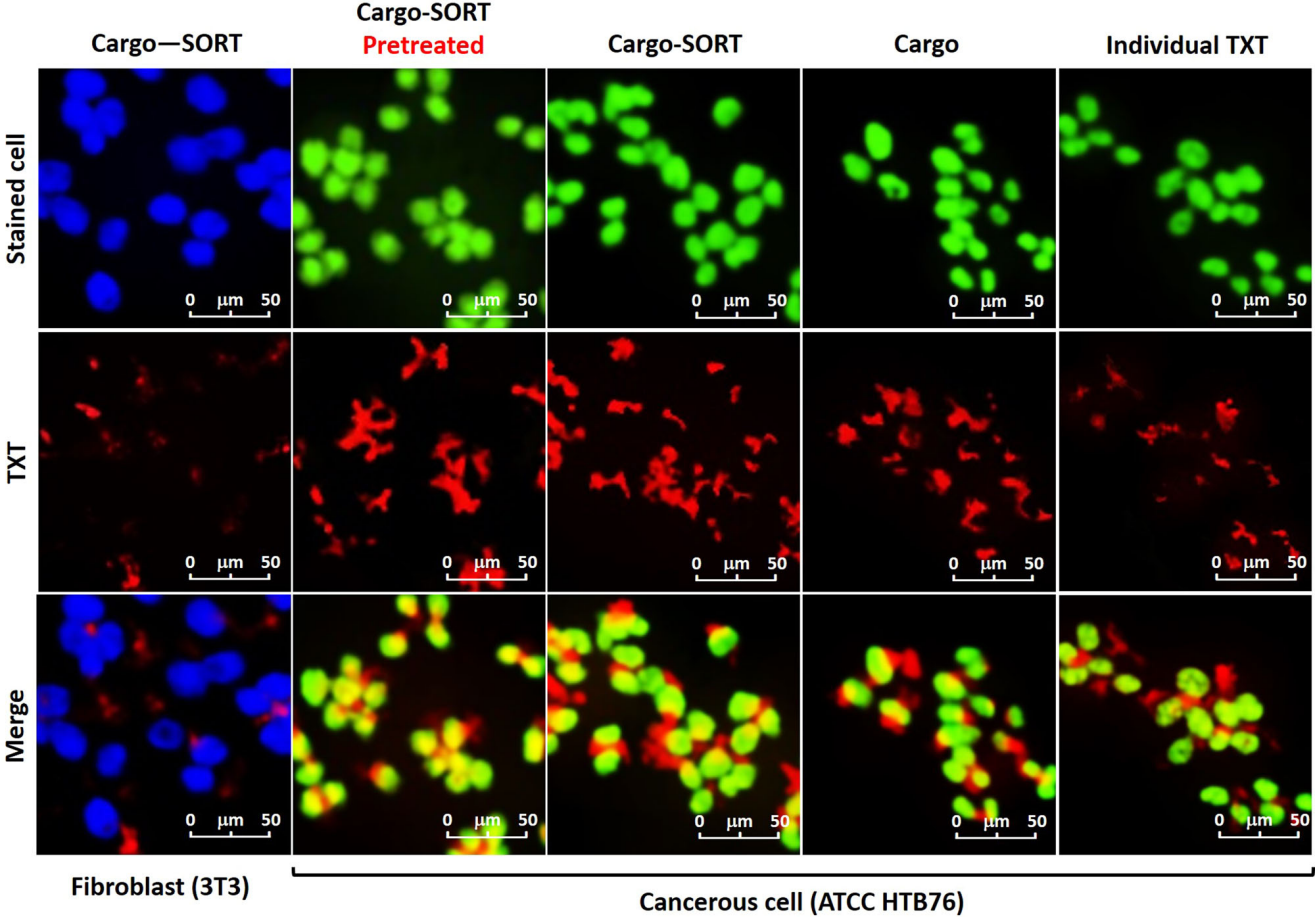
Confocal images of the subjected TXT@Fe_3_O_4_/PVA/Au nano-therapeutic to the stained cells. Green: HTB76 cancerous, and blue: NIH 3T3 fibroblast cells (10^6^ DFU), in the presence of the individual TXT, TXT@Fe_3_O_4_/PVA/Au (Cargo), and TXT@Fe_3_O_4_/PVA/Au-SORT particles (Cargo-SORT) particles. Cell staining was performed using crystal violet, and incubation was carried out at 37 °C with 95% humidity for 2 hours. Pretreatment was done with Ca(OH)_2_@Fe_3_O_4_/PVA/Au-SORT particles in the same dosage with the TXT-containing therapeutic (10 μg/mL).

To monitor the effect of SORT-expression by the HTB76 receptors on cellular uptake process, confocal microscopy with fluorescence detection was used. Moreover, we intended to investigate how this process is affected after pretreatment of the cells with Ca(OH)_2_. For this purpose, cell staining was carried out using crystal violet as a compound with PL activity at 592 nm^59^. As illustrated in Fig. 4, individual TXT (red stains) is dispersed around the target cells (green spheres) without any selectivity in the cell adhesion. Obviously, a part of this compound that is close to the cells is adhered to the cell and eventually internalizes into the cells. As can be seen in the images, there is a huge difference in the uptake manner between the HTB76 (as cancerous) and fibroblast- NIH NIH 3T3 (as normal human cells) after 2 hours incubation for the TXT@Fe_3_O_4_/PVA/Au-SORT (Cargo‒SORT) therapeutic, confirming high selectivity in cell attachment through expression of the SORT antibodies by the HTB76 cell receptors. In the confocal images, overlapping the contrasting colors (green/red or blue/red) demonstrates that the ingredients have been co-localized, and most likely the pursued drug has internalized into the stained cells. Also, the intensity of the merged colors (yellow and purple) depends on the ratio of the co-localization or internalization^60^. In the case of TXT@Fe_3_O_4_/PVA/Au particles (Cargo, not conjugated to SORT antibody), this is clearly recognized that although the internalization is enhanced than the individual TXT, there is still no significant difference of selective cell attachment with the NIH NIH 3T3 cells. While, after conjugation to the SORT antibodies (Cargo‒SORT), this is conveniently recognized that the intensity of the yellow color in the merged image has increased. Moreover, the attachment of the Cargo‒SORT particles onto the HTB76 cells is clearly verified. For the comparison between the individual TXT and Cargo, the observed enhancement in the cellular uptake process is attributed to the known interactions of nanomaterials with the living cells and also the jelly nature of the PVA matrix leading to more cell adhesion^61^. For the Cargo‒SORT particles, this is observed that a high level of internalization into the HTB76 cells has been obtained in comparison with the fibroblast- NIH NIH 3T3 cells. From this observation, the selective function of the TXT@Fe_3_O_4_/PVA/Au-SORT nano-therapeutic in cell adhesion through conjugated SORT antibody is well confirmed. Ultimately, the effect of pretreatment of the cells with Ca(OH)_2_ was investigated via subjection of the same dosage of Ca(OH)_2_@Fe_3_O_4_/PVA/Au-SORT particles (10 μg/mL) to the cells through six hours of incubation. Then, the main treatment on the target cells was performed via subjection of the TXT@Fe_3_O_4_/PVA/Au-SORT particles to the cells for 12 hours. As is seen in the images (Cargo-SORT, pretreated), no decrease in the intensity of the yellow color (internalized cargo) in comparison with the case of direct treatment (previous case) is observed. Briefly, this is concluded that the considered pretreatment has no negative influence on the function of the caov-4 receptors, which encode SORT antibody onto the ATCC HTB76 cell surfaces.

#### Evaluation of antigen-mediated cell attachment and migration inhibition potency of TXT@Fe_3_O_4_/PVA/Au-SORT nano-therapeutics

**Fig. 5:**
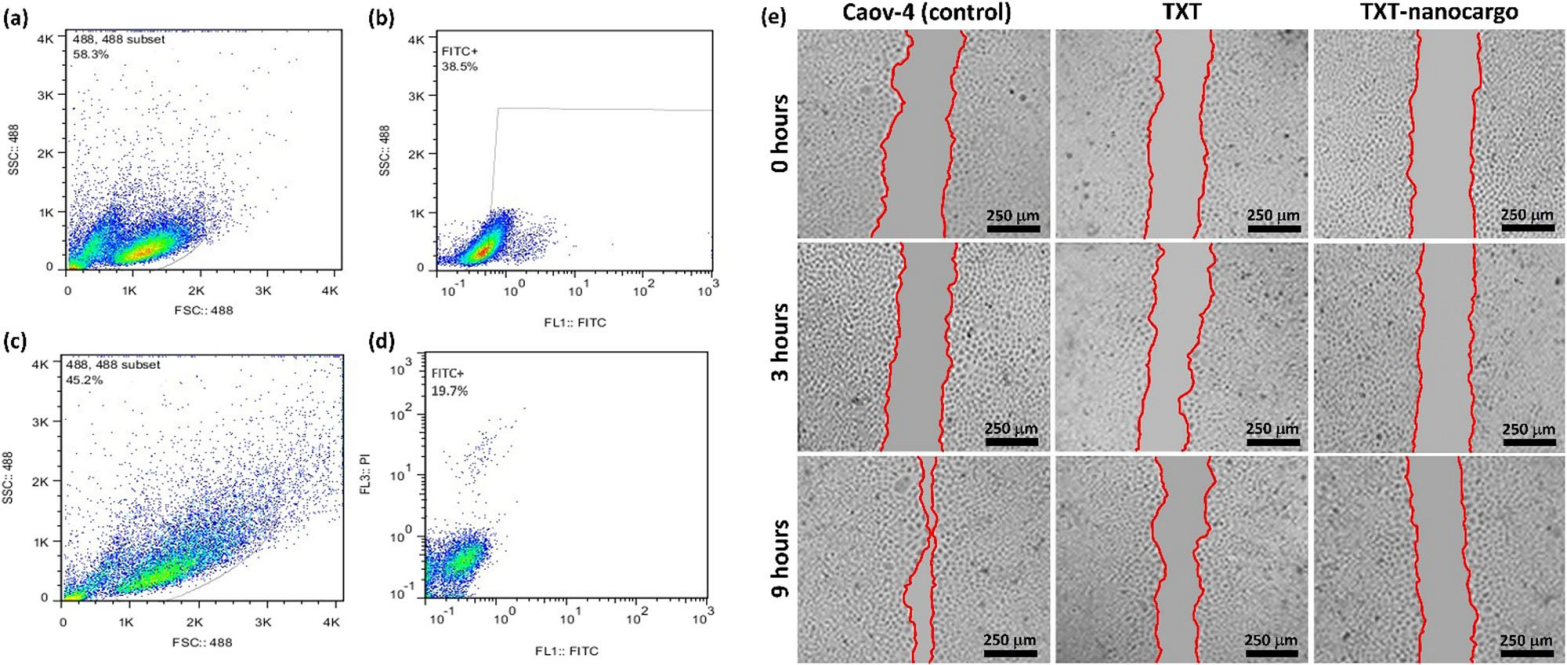
The obtained results from flow cytometry analysis and migration inhibition scratch assay. **a**–**d** Flow cytometry FSC×SSC density plots of individual SORT antibodies (**a**, **b**) and TXT@Fe_3_O_4_/PVA/Au-SORT nano-therapeutics (**c**, **d**) over caov-4 (HTB76) ovarian cancer cells, incubated at 4 °C (*n* = 3). **a**, **c** Illustrate ungated subset with 488 nm excitation, and **b**, **d** illustrate FITC-gated results. **e** Results of migration inhibition scratch assay using individual TXT and TXT@Fe_3_O_4_/PVA/Au-SORT nano-therapeutics (TXT-nanocargo) in comparison with the caov-4 control, in a nine-hour process.

HTB76 cell expression ratio of TXT@Fe_3_O_4_/PVA/Au-SORT nano-therapeutic was compared with the individual SORT, as control. For this purpose, forward- by side-scattering (FSC×SSC) density plots (488 nm subset) were prepared via flow cytometry. In this regard, trypan blue (wavelength = 660 nm) was used for cell staining as the first stage of the study. Then, the population of the stained cells was counted and considered as 100%. Afterward, the population of the living cells was counted after blocking and rinsing stages. To estimate successfully expressed SORT antibodies by caov-4 living cells, fluorescein isothiocyanate (FITC) as a secondary antibody was used. As shown in the illustrated plots in Fig. 5a, b, (53.4 ± 1.1) % cell expression has been counted after FITC-gating of the density plot, confirming that the cell expression of the sortilin 2D8-E3 antibody by caov-4 cell line is (66.0 ± 3.1) % (38.5/58.3 × 100)^59^. In Fig. 5c, d, it has been demonstrated that (43.6 ± 4.3) % (19.7/45.2 × 100) cell expression is occurred for TXT@Fe_3_O_4_/PVA/Au-SORT nano-therapeutics, confirming high selectivity in cell attachment. It should be noted that the observed decrease in the obtained results for the TXT@Fe_3_O_4_/PVA/Au-SORT nano-therapeutic in comparison with the individual SORT may originate from possible conjugation of a part of antibodies from their complementarity-determining region (CDR) to the particles. Moreover, to investigate selective expression of the TXT@Fe_3_O_4_/PVA/Au-SORT system by caov-4 cells, MCF-7 cell line was experimented as an irrelevant strain via the same procedure as caov-4. As presented in the obtained plots (shown in Figure S10, in the SI section), only (4.1 ± 2.6) % of the MCF-7 cells were successfully counted after FITC-gating process meaning that the SORT antibodies are specifically expressed by caov-4 cancerous cells.

One of the well-known in vitro techniques for screening the migration ability of the living cells and its inhibition by toxic drugs is the scratch assay experiment^62^. To evaluate the potential of the prepared TXT@Fe_3_O_4_/PVA/Au-SORT nano-therapeutics to inhibit the migration of caov-4 cells, a time-dependent experiment was performed in a range of 0–9 hours, and the obtained results have been exhibited in Fig. 5e. As shown, in the case of the individual caov-4 control, a significant contraction (*P* < 0.01) was observed by the living cells during the 9 hours incubation, whereas no considerable migration was seen within treatment with the individual TXT and TXT@Fe_3_O_4_/PVA/Au-SORT nano-therapeutics. With the same dose of the individual TXT and TXT@Fe_3_O_4_/PVA/Au-SORT, almost the same results were obtained while only 13.3 wt% of the TXT@Fe_3_O_4_/PVA/Au-SORT system is formed by TXT. The results depict that the presented TXT@Fe_3_O_4_/PVA/Au-SORT (containing 13.3 wt% of TXT) has a great inhibition effect on the migration ability of the cancer cells along with the high degree of biosafety than the sole TXT.

#### In vitro bioassay of cell mortality upon exposure to TXT@Fe_3_O_4_/PVA/Au-SORT by methyl tetrazolium test

**Fig. 6:**
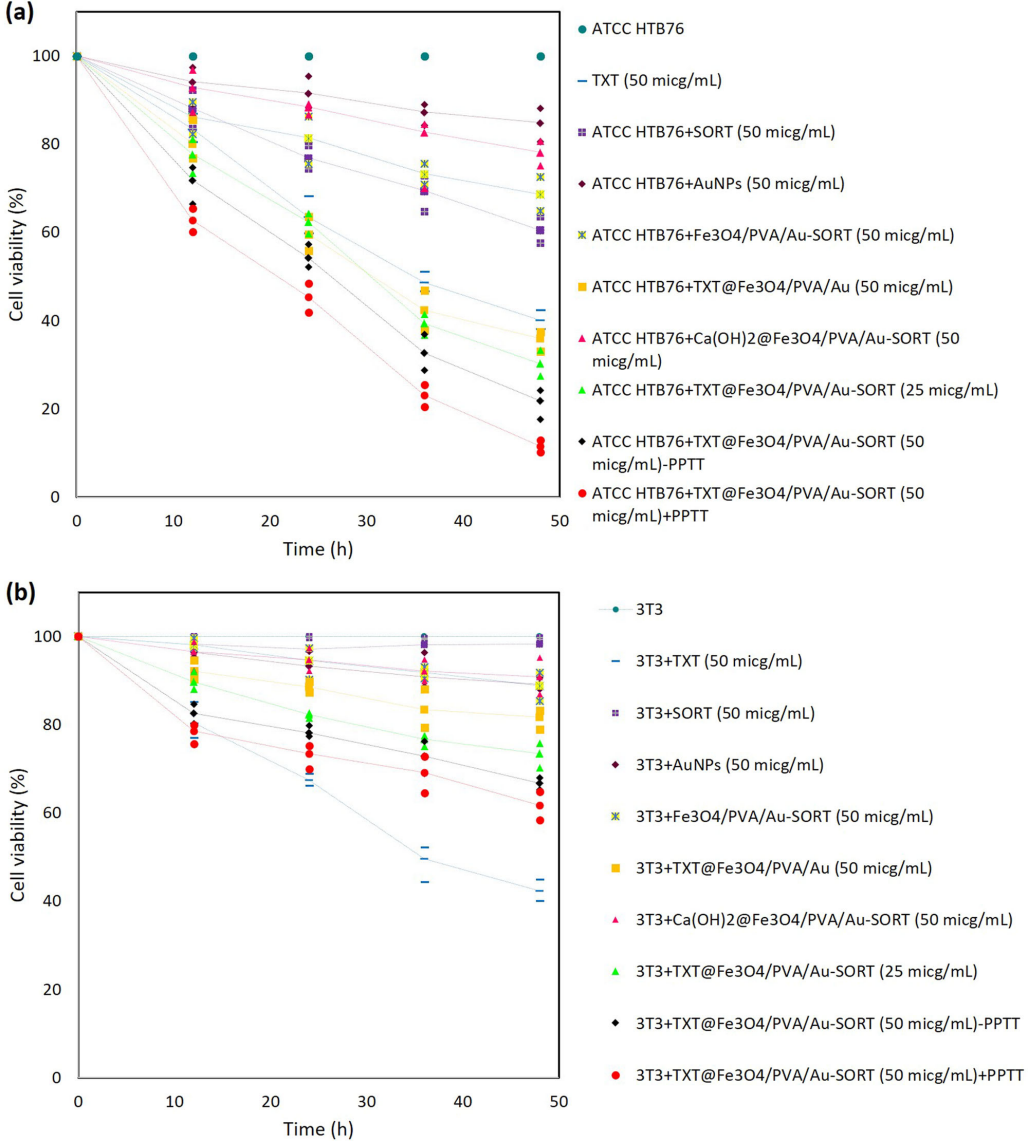
MTT assay results of TXT@Fe_3_O_4_/PVA/Au-SORT nano-therapeutic over the cancerous and normal cell strains. The doses of 25 and 50 μg/mL of TXT@Fe_3_O_4_/PVA/Au-SORT particles were subjected to; **a** caov-4 (HTB76) ovarian cancer cells, and **b** NIH 3T3 fibroblast human cells, in comparison with a dose of 50 μg/mL. +PPTT and –PPTT stand for the presence and absence of the green LED light (7 W, 526 nm), respectively. Irradiation was performed for 10 minutes every 2 hours. The incubation was carried out at 37 °C with 95% humidity.

To investigate enhanced cytotoxicity of TXT along with selectivity through administration of TXT@Fe_3_O_4_/PVA/Au-SORT nano-therapeutic, methyl tetrazolium (MTT) experiment was carried out^63^. For this aim, HTB76 (cancerous) and NIH NIH 3T3 (fibroblast) cells were cultivated and their growth rates were screened in the absence and presence of the TXT@Fe_3_O_4_/PVA/Au-SORT particles and also TXT, sortilin 2D8-E3 antibody (SORT), AuNPs, Fe_3_O_4_/PVA/Au-SORT, TXT@Fe_3_O_4_/PVA/Au, and Ca(OH)_2_@Fe_3_O_4_/PVA/Au-SORT particles, with the same concentration (50 μg/mL) as the controls. For the TXT@Fe_3_O_4_/PVA/Au-SORT particles as the final product, two dosages including 25 and 50 μg/mL were experimented under typical and PPTT conditions. The PPTT condition was provided by a green LED light source with 7 W power and a specified wavelength of ca. 526 nm^57^. The cell killing profile was screened in a 48-hour process for each condition. As shown in Fig. 6, there is no significant difference of growth inhibition by the individual TXT between two cell lines, confirming high levels of negative side effects of the sole TXT^57,59^. While with a quick look at the dot-graphs, this is figured out that TXT has selectively affected the HTB76 cells through administration of TXT@Fe_3_O_4_/PVA/Au-SORT particles.

As is observed, 50 μg/mL of TXT@Fe_3_O_4_/PVA/Au-SORT particles have caused (88.5 ± 9.2) % of HTB76 cell killing, under PPTT condition (+PPTT), whereas this value is (38.3 ± 3.8) % for NIH 3T3 under the same condition. At the administrated dosage of 50 μg/mL, the efficiency of PPTT-triggered release of TXT is also distinguished, where (78.2 ± 8.6) % growth inhibition is observed in the absence of LED irradiation (-PPTT), for the HTB76 cells over 48 hours. As the pretreatment agent, the same dosage of the Ca(OH)_2_@Fe_3_O_4_/PVA/Au-SORT particles was experimented under the PPTT conditions. As is seen, cell death has been started upon PPTT-release of Ca(OH)_2_, where (21.8 ± 2.1) % and (9.1 ± 0.8) % cell death have been observed on the HTB76 and NIH NIH 3T3 cells, respectively, over 48 hours incubation. From this observation, we concluded that the low dosages of Ca(OH)_2_@Fe_3_O_4_/PVA/Au-SORT particles could selectively cause a partial degradation of the cancerous cells in the massed tumor, resulting in a more penetrable mass before administration of the main therapeutic cargo. The remarkable role of the conjugated SORT antibody to the nanostructure is highlighted from the comparison of the growth inhibition values between TXT@Fe_3_O_4_/PVA/Au and TXT@Fe_3_O_4_/PVA/Au-SORT particles, with the same dosage (50 μg/mL). As is observed, a cell killing value of (64.1 ± 6.3) % has been obtained by TXT@Fe_3_O_4_/PVA/Au particles (on HTB76 cells, during 48 hours), which has a significant difference with (88.5 ± 9.2) % related to the TXT@Fe_3_O_4_/PVA/Au-SORT particles. This difference is attributed to the role of the SORT antibody. This value has been reduced to (18.3 ± 0.7) % for NIH NIH 3T3, most likely due to the absence of the acidic environment and subsequently less release of TXT inside the fibroblast cells. Moreover, the important role of the conjugated SORT antibody can be recognized from the cell killing value for the individual SORT on both cell lines. This is seen that (39.5 ± 4.9) % cell killing has been obtained by the sole SORT on HTB76 cells during 48 hours. Whereas it has such a partial effect on the NIH NIH 3T3 cells. Moreover, the effectiveness of the AuNPs has been individually monitored revealing that minor cell killing effect is obtained by this ingredient. Figure 6 also reveals the rest data of the MTT assay test related to 12, 24, and 36 hours.

#### Ex vivo study on pretreated tumors with Ca(OH)_2_@Fe_3_O_4_/PVA/Au-SORT nano-therapeutics

In order to investigate the efficacy of administration of the low dosages of Ca(OH)_2_ on tumor swelling and pre-degradation of the mass tissue of tumor, Ca(OH)_2_@Fe_3_O_4_/PVA/Au-SORT with the same configuration to TXT@Fe_3_O_4_/PVA/Au-SORT nano-therapeutic was intravenously injected to the mice. For this purpose, a seven-day treatment was applied on two groups of tumor-bearing mice, considering the magnetic direction and PPTT conditions for the controlled release of Ca(OH)_2_ in the tumor tissue. In this regard, six mice were divided into a couple of three-member groups; the first group as a control without any treatment with Ca(OH)_2_@Fe_3_O_4_/PVA/Au-SORT particles only received blank saline per each injection. The second one, as the target society received Ca(OH)_2_@Fe_3_O_4_/PVA/Au-SORT particles (50 mg/kg/day in 100 μL saline) from the tail vein for two times (on first and third days), during the seven days pretreatment period. The magnetic direction and PPTT conditions were applied via exposure of the mice to an external magnetic field (1 Tesla, 3 hours per day), and NIR laser irradiation (808 nm laser, 1.5 W/cm^2^, 5-mm spot diameter), 12 hours post-injection^64^. Finally, the mice were sacrificed on the eighth day, and the organs with the tumor were excised and evaluated.

**Fig. 7:**
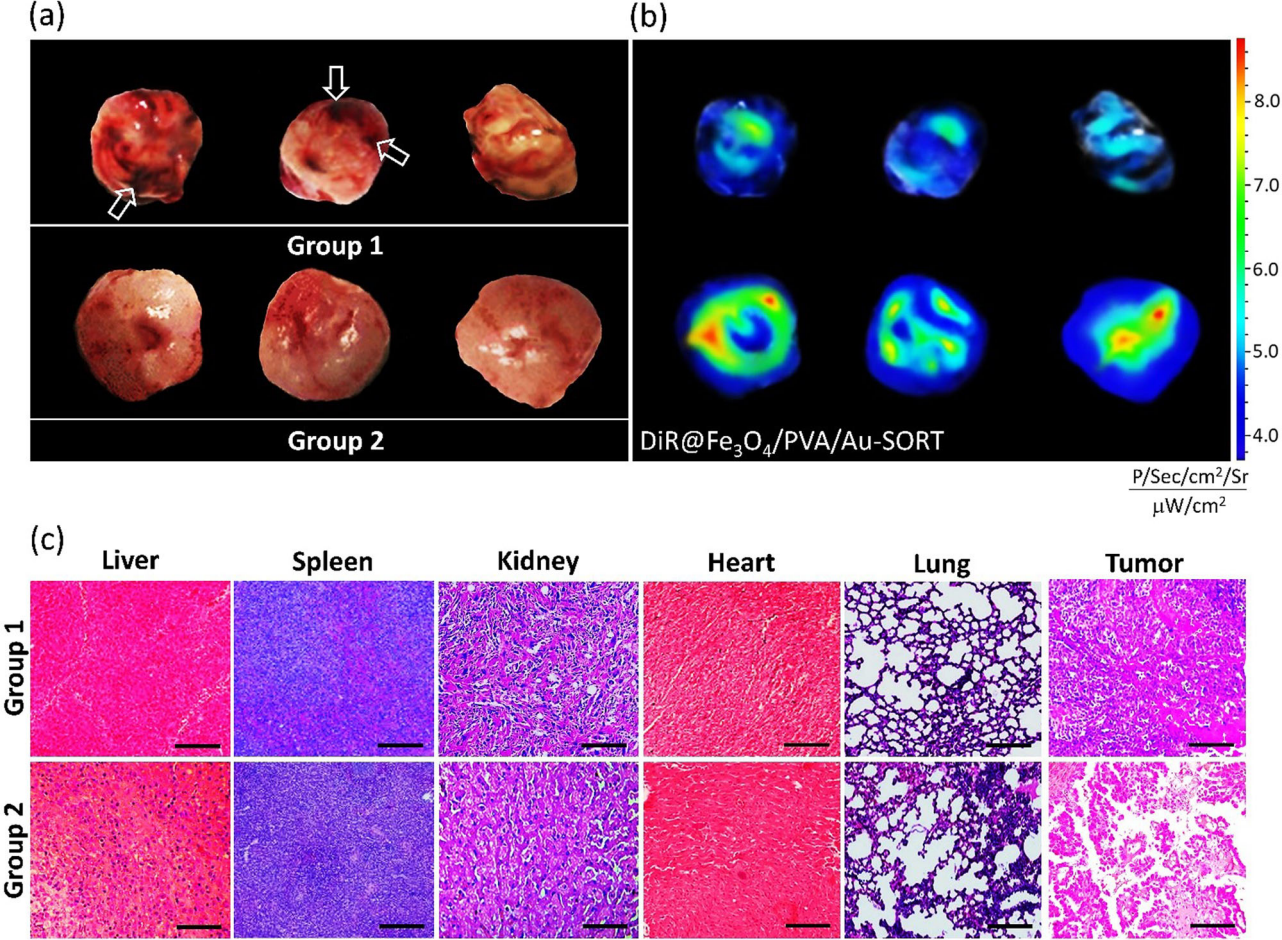
Ex vivo digital photos, fluorescence, and histopathological images of the tumor mass. **a** Ex vivo digital and **b** fluorescence images, obtained via injection of 50 mg/kg/day of DiR@Fe_3_O_4_/PVA/Au-SORT in 100 μL saline, on the sixth day of pretreatment period. **c** H&E histopathological images of tumor masses and untargeted organs in the mice of two groups (all scale bars are 100 μm).

As illustrated in Fig. 7a, pretreatment results have revealed that a softer and more penetrable mass is obtained, which seems to be more responsive to the immunological ingredients like antibody. In the prepared digital images, dark sections (marked by white arrows) show the massed parts in the tumor. To evaluate the tumor penetration ratio after the pretreatment program, tetramethylindocarbocyanine iodide (DiR) was incorporated into the Fe_3_O_4_/PVA/Au-SORT drug nano-carrier instead of the TXT via a similar loading procedure (see section 4.3.10)^65^. On the sixth day of the pretreatment program, DiR@Fe_3_O_4_/PVA/Au-SORT (50 mg/kg/day in 100 μL saline) was injected into the mice of two groups according to the same protocol applied for the Ca(OH)_2_@Fe_3_O_4_/PVA/Au-SORT. The DiR fluorescence distribution was studied using a Maestro in vivo imaging system, as described in Fig. 7b. Further, a thin slice of the tumor tissue with ca. 4-μm width was prepared and fixed in a mixture of picric acid, formaldehyde and glacial acetic acid. Then, the slices were stained with H&E (Hematoxylin and Eosin) and studied by a light microscope^66^. As is observed in Fig. 7c, the tissue of the excised tumor from the mice body related to the second group (treated with Ca(OH)_2_@Fe_3_O_4_/PVA/Au-SORT) includes more cracks proving damages to the massed tumor, while no crack is seen for the control mice (group 1). Also, in the image related to the treated mice, this is clearly seen that the color has been faded, confirming degradation of the massed tumor through pretreatment by Ca(OH)_2_. Moreover, to investigate possible damages to other healthy organs of the body during pretreatment by Ca(OH)_2_, biosafety was controlled on the samples of the sliced organs in the same manner. As is observed in the H&E pathological images, there is no vestige of degradation in the healthy tissues, including liver, spleen, kidney, heart, and lung, excised from the mice body of the second group.

#### Screening of DNA damaging induced by TXT@Fe_3_O_4_/PVA/Au-SORT nano-therapeutic (γH2AX phosphorylation experiment)

**Fig. 8:**
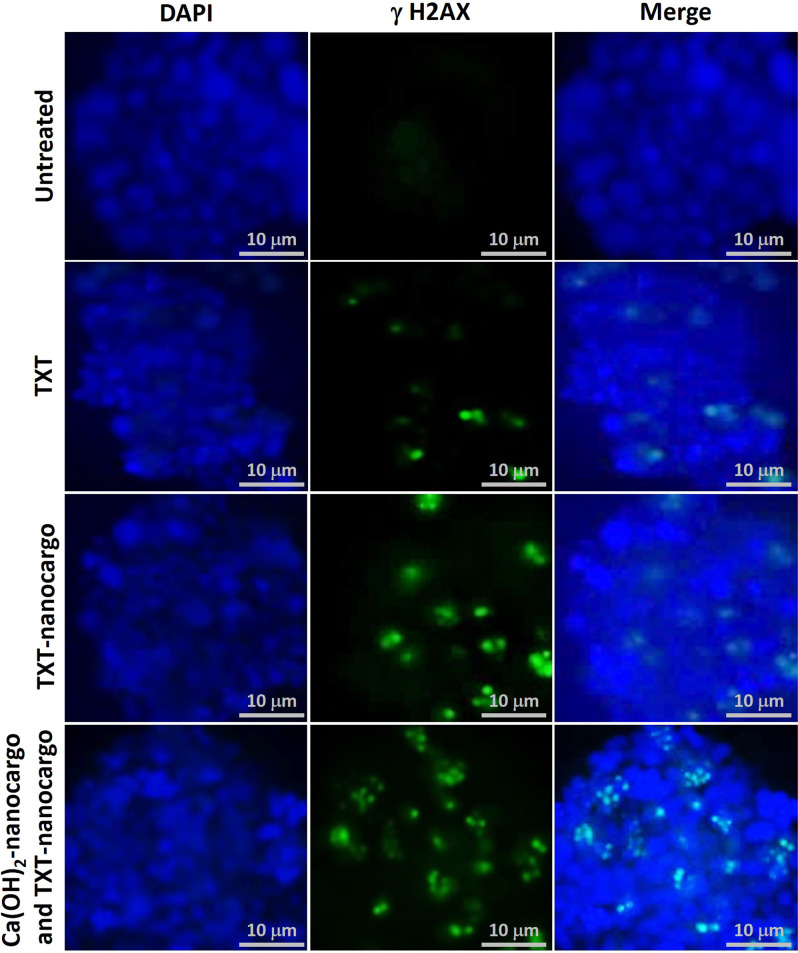
γH2AFX phosphorylation confocal images of DAPI stained tumor mass. Green: phospho-γH2AFX, and blue: DAPI counterstain of the nuclei to provide caov-4 cancer tumor cells), in the presence of the individual TXT, TXT@Fe_3_O_4_/PVA/Au (TXT-Cargo), and Ca(OH)_2_@Fe_3_O_4_/PVA/Au-SORT (Ca(OH)_2_-nanocargo) particles. The green spots indicate phospho-cH2AFX-positive caov-4 cells in the images of tumors harvested 48 hours post injection. Images are enhanced +10 brightness and +30 contrast to aid in visual clarity.

To investigate how DNA of the caov-4 cells are damaged by the subjected Ca(OH)_2_@Fe_3_O_4_/PVA/Au-SORT and TXT@Fe_3_O_4_/PVA/Au-SORT nanocargoes, we stained tumor sections for phosphorylated *γ*H2AFX^67^. Concisely, *γ*H2AFX is activated (phosphorylated) through attachment to the double-strand DNA fragments even at very early stages of damage. As illustrated in Fig. 8, phospho-*γ*H2AFX were detected (green spots) 48 hours post-injection of TXT, TXT@Fe_3_O_4_/PVA/Au-SORT (TXT-nanocargo), and Ca(OH)_2_@Fe_3_O_4_/PVA/Au-SORT (Ca(OH)_2_-nanocargo) then TXT@Fe_3_O_4_/PVA/Au-SORT nanocargoes. In the obtained confocal images, two factors should be noticed; 1. The number of the green spots which is proportional to the damaged cells, and 2. The intensity of the green light through the appeared spots that origins from the damage rate. As is observed in the representative images, both number and intensity of the green spots were higher after consecutive treatment by Ca(OH)_2_@Fe_3_O_4_/PVA/Au-SORT then TXT@Fe_3_O_4_/PVA/Au-SORT nanocargoes, corroborating severe damage to the apoptotic cells. In the same line, a bit more damage is observed for the TXT-nanocargo compared to the individual TXT. The ovary tumor nuclei was stained by 4′,6-diamidino-2-phenylindole (DAPI) that appeared in blue in the provided images. These data have revealed that caov-4 cancer exhibit a DNA damage response to Ca(OH)_2_@Fe_3_O_4_/PVA/Au-SORT and TXT@Fe_3_O_4_/PVA/Au-SORT nanocargoes, while this response is lower in the case of the individual TXT with the same dosage as nanocargo.

#### In vivo studies on tumor pretreatment by Ca(OH)_2_@Fe_3_O_4_/PVA/Au-SORT and treatment by TXT@Fe_3_O_4_/PVA/Au-SORT nano-therapeutics

**Fig. 9:**
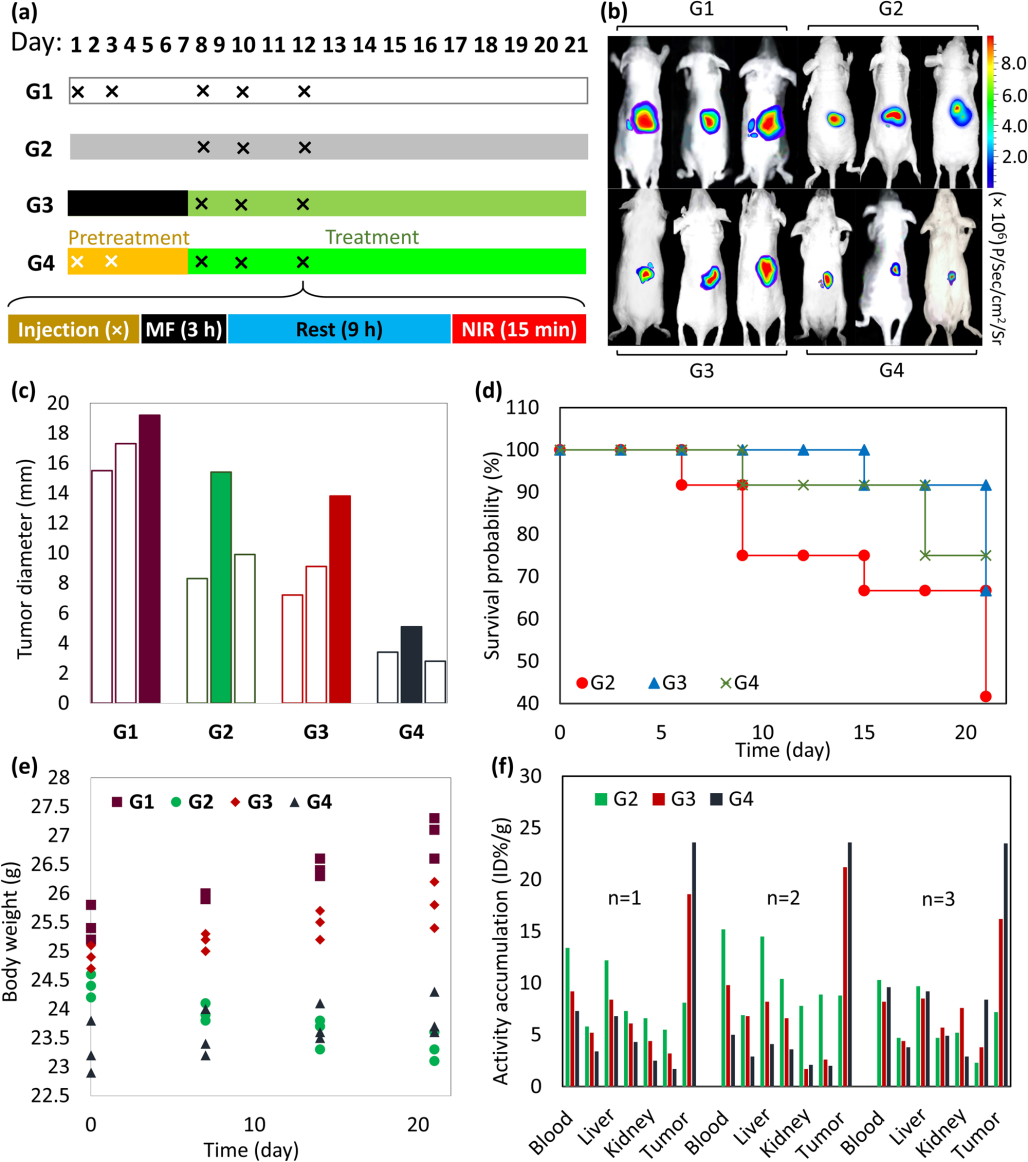
Presentation of the designed pretreatment and treatment plans, and the obtained results from bioluminescence imaging, survival, tumor diameter, mice body weight, and biodistribution evaluations. **a** Schematic presentation of designed pretreatment and treatment plan, executed by selective delivery of Ca(OH)_2_@Fe_3_O_4_/PVA/Au-SORT and TXT@Fe_3_O_4_/PVA/Au-SORT nano-therapeutic. *G1*: Three mice as control received blank saline, *G2*: three mice as control received TXT, *G3*: three mice as control received TXT@Fe_3_O_4_/PVA/Au-SORT (50 mg/kg/day in 100 μL saline) in a time range of 8–21th, and *G4*: three mice as target society received Ca(OH)_2_@Fe_3_O_4_/PVA/Au-SORT (50 mg/kg/day) within the first week, and then TXT@Fe_3_O_4_/PVA/Au-SORT in the same procedure as *G3*. Injection from tail vein (×), magnetic field (MF), and NIR: 808 nm laser, 1.5 W/cm^2^, 5-mm spot diameter, for 15 min. D-luciferin (150 μg/kg) was intraperitoneally injected on 20th day, and the mice were euthanized after 21 days. **b** Bioluminescence images of the experimented mice, prepared on 21th of the study. **c** Tumor diameter diagram of the nine experimented mice. **d** Survival curves related to twelve mice in each group (*G2*-*G4*) during a 21-day treatment program (it should be stated that no statistical data were investigated for this experiment, since the mice society population for each time is 36. Hence, it is not justified from economic and ethical aspects to repeat the experiment for three times). **e** Dot-plot graphs of the measured body weight related to the experimented mice (*G1*–*G4*), during a 21-day in vivo study. **f** Biodistribution diagram for the mice organs of *G2*–*G4*, after 24-h treatment [The values are reported as a percentage of the injected dose per gram of each tissue (%ID/g)].].

The efficacy of pretreatment by Ca(OH)_2_ has been studied with luciferase-expressing caov-4 tumor-bearing cells using 9–11 weeks-old female mice. For this purpose, subcutaneous injection of luciferase-expressing HTB76 cells (2 × 10^6^) was done into the mouse flank. Totally, twelve mice were selected and divided into four three-member groups, including three mice as a control without any treatment (*G1*), three mice treated by individual TXT (*G2*), three mice for 14 days treatment only with TXT@Fe_3_O_4_/PVA/Au-SORT nano-therapeutic (*G3*), and the rest for a 7-day pretreatment with Ca(OH)_2_@Fe_3_O_4_/PVA/Au-SORT particles plus a 14-day treatment with TXT@Fe_3_O_4_/PVA/Au-SORT nano-therapeutic (*G4*). For the third and fourth groups, magnetic direction and PPTT conditions have been applied according to a similar procedure described in the previous section. Tumor-bearing process was performed for 30 days, and the pretreatment and treatment periods were started with the injection of the first dose of Ca(OH)_2_@Fe_3_O_4_/PVA/Au-SORT and TXT@Fe_3_O_4_/PVA/Au-SORT nano-therapeutic to the mice of *G3* and *G4*, respectively. For the treatment of the mice of *G3*, the injection of TXT@Fe_3_O_4_/PVA/Au-SORT nano-therapeutic (50 mg/kg/day in 100 μL saline) was carried out on the first, third, and fifth days of the period, as our previous protocol^57^. For the fourth group, the injection of a suspension of Ca(OH)_2_@Fe_3_O_4_/PVA/Au-SORT particles was done on the first and third days of the 7-day pretreatment, then TXT@Fe_3_O_4_/PVA/Au-SORT nano-therapeutic was injected on the first, third, and fifth days of the 14-day treatment period (Fig. 9a). The mice of *G3* and *G4* were exposed to an external magnetic field (1 Tesla) for three hours, immediately post-injection, and also NIR laser irradiation (808 nm laser, 1.5 W/cm^2^, 5-mm spot diameter) for 15 min, 12 hours post-injection. The tumor penetrating capability and the effectiveness of the drug-loaded Fe_3_O_4_/PVA/Au-SORT nano-therapeutic was investigated by bioluminescence imaging through injection of D-luciferin (150 μg/kg). The luminescence intensity correlates with the size of the formed tumor^57,59,68^. As is observed in the images shown in Fig. 9b, c, the mice of *G3* treated with TXT@Fe_3_O_4_/PVA/Au-SORT nano-therapeutics for 14 days exhibited tumor growth inhibition by (42.1 ± 3.3) %, whereas this value is (78.3 ± 1.2) % for the mice of *G4*, which selectively received Ca(OH)_2_ as pretreatment for a week and then the main treatment by TXT@Fe_3_O_4_/PVA/Au-SORT nano-therapeutics. In the case of treatment by the individual TXT (*G2*), (35.4 ± 3.7) % of tumor growth has been inhibited by TXT in the same doses as TXT@Fe_3_O_4_/PVA/Au-SORT nano-therapeutics (*G3*). This equal activity of the individual TXT and TXT@Fe_3_O_4_/PVA/Au-SORT nano-therapeutics well confirms the efficiency of the designed carrier system because only ca. 13 wt% of the total weight in TXT@Fe_3_O_4_/PVA/Au-SORT is formed by TXT. In the other words, the same antitumor efficacy has been obtained by TXT@Fe_3_O_4_/PVA/Au-SORT, which includes high degrees of biosafety than the individual TXT. The tumor diameter diagram for 12 mice after excising the tumors is shown in Fig. 9c. This observation well verifies that tumor penetration is significantly enhanced via implementation of a short-period pretreatment using Ca(OH)_2_, resulting in better expression of SORT antibodies by the caov-4 receptors and enhanced antibody-mediated internalization of the therapeutic cargo. The survival proportion of each group was also investigated, where the adverse effects of the individual TXT in *G2* led to high mortality (60%) during 21 days of treatment. As presented in the curves (Fig. 9d), the survival rate in *G4* was 75%, which is higher than the other groups. However, no significant difference was observed between the G3 and G4, which corroborates high biosafety of the Fe_3_O_4_/PVA/Au-SORT carrier system and the applied method.

The weight values of the experimented mice were also measured during a 21-day in vivo study. As Fig. 9e presents, the mice of *G1* gained in three weeks due to rapid tumor growth as was expected because no treatment was performed on them. In contrast, the mice of *G2* have shown a tangible weight loss, which can be attributed to the lack of biosafety and tissue degradation through receiving the TXT as a cytotoxic medication. In *G3*, since the TXT has been selectively delivered to the tumor tissue, high degree of biosafety was expected. The upward trend in the bodyweight of the mice of *G3* corroborates this claim. The bodyweight of the mice of *G4* had no significant change during the pretreatment and treatment programs in comparison with the mice of *G1* and *G2*. Based on these data, it is concluded that tumor growth has been greatly inhibited via the presented strategy. Moreover, high grade of biosafety through selective delivery of Ca(OH)_2_ and TXT can be verified by these results, as any possible damages to the healthy tissues can lead to degradation of the tissues, and consequently weight loss. Figure 9f, represents biodistribution of the neat DiR (for *G2*) and DiR@Fe_3_O_4_/PVA/Au-SORT (for *G3* and *G4*) in the important organs. As is observed in the chart, the obtained results well confirm that more aggregation of the particles occurred in the pretreated tumor with Ca(OH)_2_@Fe_3_O_4_/PVA/Au-SORT nano-therapeutics (*G4*), in comparison with *G2* and *G3*. These results also confirm the selective function of the Fe_3_O_4_/PVA/Au-SORT drug carrier and the efficiency of the presented method.

## Conclusion

To overcome impermeability of the aged tumor masses, an effective method has been suggested. In summary, an advanced drug delivery system constructed of Fe_3_O_4_ magnetic nanoparticles, PVA shell, and AuNPs (incorporated into the PVA) has been applied for selective delivery of Ca(OH)_2_ and TXT to the cancer tumor. To enhance biological targeting of the caov-4 cancerous cells, the particle surfaces have been functionalized with human IgG1 SORT monoclonal antibody. To have high control in the drug release process, the PPTT effect of the AuNPs under irradiation of the green LED (ca. 526 nm) and NIR light sources has been used. The in vitro cellular experiments have confirmed high growth inhibition potency (ca. 89%) for TXT@Fe_3_O_4_/PVA/Au-SORT nano-therapeutic compared to the individual TXT (with the same dosage) and other controls. Confocal microscopy has demonstrated high selectivity in cell attachment and internalization for the prepared cargo. As well, ca. 47% cell expression by caov-4 cells has been confirmed for TXT@Fe_3_O_4_/PVA/Au-SORT nano-therapeutic. In the in vivo experiment plan, a 21-day treatment program has been executed on the mice, as follows; during the first week, pretreatment of the formed tumor mass was performed via injection of two dosages of Ca(OH)_2_@Fe_3_O_4_/PVA/Au-SORT, and further magnetic direction and NIR irradiation. Then, a 14-day treatment period with TXT@Fe_3_O_4_/PVA/Au-SORT nano-therapeutic was immediately implemented. The efficacy of seven days pretreatment with Ca(OH)_2_@Fe_3_O_4_/PVA/Au-SORT has been well corroborated by the fluorescence imaging, where more tumor penetration ratio has been observed in comparison with the controls. Also, H&E histopathological imaging has confirmed the presence of necrosis degradation in the pretreated tumor tissue with Ca(OH)_2_. The bioluminescence imaging on the mice has confirmed tumor growth inhibition by ca. 78% during a 7-day pretreatment plus 14 days treatment programs. Due to showing high values of the anti-tumor properties and biosafety, the presented pretreatment strategy is suggested to have more effective treatment for the aged tumor tissues.

## Methods

### Materials and equipment

All chemicals and instruments used in this study have been listed in Tables S1 and S2, the SI file.

### Practical procedure

#### Synthesis of Fe_3_O_4_ nanoparticles

In a glass round-bottom flask (100 mL), FeCl_2_.4H_2_O (5.0 mmol), FeCl_3_.6H_2_O (6.0 mmol), and deionized water (50 mL) were well mixed at 80 °C, via magnetic stirring. Then, NH_4_OH (8.0 mL, 25 wt%) was dropwise added to the solution during 30 min, under N_2_ atmosphere. After completion of the addition, the mixture was stirred for an additional 2 h, at the same condition. Ultimately, the temperature was reduced to room temperature, and the formed nanoparticles were magnetically collected by holding an external magnet at the bottom of the flask. The particles were rinsed with distilled water and ethanol several times and dried in an oven at 60 °C.

#### Preparation of Fe_3_O_4_/SiO_2_ nanoparticles

In a glass round-bottom flask (100 mL), Fe_3_O_4_ nanoparticles (1.0 g) were mixed with deionized water (10 mL) and well dispersed via ultrasonication (50 KHz, 100 W L^−1^), for 15 min. Next, EtOH (5.0 mL), PEG-300 (5.0 mL) and NH_4_OH solution (1.0 mL, 25 wt%) were added to the mixture and stirred for 10 min, at room temperature. Afterward, tetraethyl orthosilicate (2.0 mL) was dropwise added into the flask during the stirring for 1 h. Eventually, Fe_3_O_4_/SiO_2_ nanoparticles were magnetically collected and washed several times with ethanol and dried in an oven at 60 °C.

#### Preparation of Fe_3_O_4_/SiO_2_/PVA nanoparticles

In a glass round-bottom flask (100 mL), Fe_3_O_4_/SiO_2_ nanoparticles (0.5 g) were dispersed in deionized water (10 mL), and several portions of PVA-72000 (1.0 g) were added to the flask during ultrasonication (50 KHz, 100 W L^−1^) for 30 min, at 80 °C. Then, the mixture was stirred for 2 h, under gentle reflux conditions. Ultimately, Fe_3_O_4_/SiO_2_/PVA nanoparticles were magnetically collected, rinsed, and dried according to the same procedure in the previous stage.

#### Preparation of Fe_3_O_4_/PVA-CPS nanoparticles

In a glass round-bottom flask (100 mL), Fe_3_O_4_/SiO_2_/PVA nanoparticles (0.5 g) were dispersed in EtOH (10 mL), and a solution of 3-CPTMS (5.0 mL) in toluene (5.0 mL) was dropwise added to the mixture during the ultrasonication, in 30 min. Then, the mixture was stirred for 2 h, under gentle reflux conditions. Ultimately, Fe_3_O_4_/PVA-CPS nanoparticles were magnetically collected, rinsed, and dried according to the same procedure in the previous stage.

#### Preparation of TXT@Fe_3_O_4_/PVA-CPS nanoparticles

In a glass tube (13 by 100 mm, equipped with a threaded cap), Fe_3_O_4_/PVA-CPS nanoparticles (0.1 g) were dispersed in a solution of TXT (5.0 mL, 0.5 M in EtOH), and the temperature was increased to 37 °C, and the whole tube was insulated with glass wool. The content of the test tube was shaken for 4 h. Eventually, TXT@Fe_3_O_4_/PVA-CPS nanoparticles were magnetically collected, rinsed with deionized water, and dried in a freeze-dryer for 48 h.

#### Preparation of gold nanoparticles

In a glass round-bottom flask (25 mL), deionized water (16.2 mL), tetracholoroauric acid (0.2 mL, 5.0 mM in water), and sodium borohydride (fresh, 0.4 mL, 1.0 mM, in water) were mixed via magnetic stirring, at room temperature. Then, trisodium citrate (0.2 mL, 5.0 mM, in water) and additional portion of tetracholoroauric acid (0.5 mL, 5.0 mM in water) were added. Afterward, the flask was exposed to LED light (1 W, 414 nm), and stirring was continued for 16 h at room temperature. Finally, the content was centrifuged (4 K rpm, 10 min), until red color particles were precipitated at the end of a falcon. The particles were then rinsed with deionized water to remove the excess salts.

#### Preparation of TXT@Fe_3_O_4_/PVA/Au-SORT nano-therapeutic

In a glass round-bottom flask (25 mL), TXT@Fe_3_O_4_/PVA-CPS nanoparticles (0.1 g) and the prepared gold nanoparticles (2.0 mL, 0.15 mM) were dispersed in 4.0 mL of phosphate buffer saline (PBS, 0.1 M, pH = 8), and the mixture was stirred at 4 °C. Afterward, sortilin 2D8-E3 mAb (10 μL, 50 μg/mL) was added, and stirring was continued for 2 h, at the same conditions. Ultimately, TXT@Fe_3_O_4_/PVA/Au-SORT particles were magnetically collected, rinsed with cold deionized water, and dried in a freeze drier for 48 h.

#### Preparation of Ca(OH)_2_@Fe_3_O_4_/PVA/Au-SORT

For this purpose, the same procedure as section 2.2.5 was followed with this difference; a solution of Ca(OH)_2_ (5.0 mL, 0.05 M in water) was subjected to the Fe_3_O_4_/PVA-CPS nanoparticles instead of TXT.

### Drug content and release experiments

#### Standard solutions

In a 100 mL volumetric balon joje, TXT (50.25 mg) was put and dissolved in 5.0 mL of EtOH, with the help of ultrasonication. Then, the volume of the solution was raised to 100 mL with EtOH (25 °C). Subsequently, the standard solutions with 5, 10, 15, 25, and 30 ppm were prepared via dilution of the stock solution (500 ppm) with the PBS (0.1 M, pH = 7.3) in the volumetric joje balloons, as follows; 1.0 to 100 mL, 2.0 to 100 mL, 1.5 to 50 mL, 5.0 to 100 mL, and 3.0 to 50 mL.

#### Sample preparation

In a glass beaker (50 mL), TXT@Fe_3_O_4_/PVA/Au-SORT nanoparticles (0.05 g) were grinded via ball-milling (25 Hz, 10 min) and dispersed in dimethylsulfoxide (DMSO) (25.0 mL) via ultrasonication (50 KHz, 100 W L^−1^), at 40 °C for 1 h. Then, the mixture was vigorously stirred for an additional 1 h at the same condition. Next, the particles were magnetically separated and the rest was filtrated by paper filters to obtain a clear solution. The obtained solution was diluted (2.0 mL to 25.0 mL) with ethanol and studied by UV-vis spectroscopy.

### Cellular experiments

#### Confocal microscopy

In a sterilized glass tube (13 by 100 mm), TXT@Fe_3_O_4_/PVA/Au-SORT nanoparticles (0.05 mg) were dispersed in Dulbecco’s modified eagle medium (DMEM) (5.0 mL), via ultrasonication (50 KHz, 100 W L^−1^) for 10 min. Then, HTB76 cells (10^6^ CFU) were added into the tube, and the content was incubated for 2 h, at 37 °C and 95% humidity. This procedure was repeated for NIH 3T3 fibroblast cells at the same conditions. Finally, the cells were dried at room temperature and stained with crystal violet (C_25_N_3_H_30_Cl) in DMEM (1% v/v, four drops) and a drop of Lugol’s solution^57^.

#### Flow cytometry

In a sterilized glass tube (13 by 100 mm), HTB76 cells (10^6^ CFU) were stained with trypan blue (1% v/v, four drops) in PBS (0.1 M, pH = 6.8), diluted with DMEM and Lugol’s solution. Next, the cells were rinsed with PBS and recollected via centrifugation (2 K rpm, 5 min). Then, cell counting was carried out via excitation at 488 nm. Afterward, rinsing with antibody (10 μg/mL) was performed in sheep’s serum (5 wt%) at 4 °C for 30 min, to clock unspecific sites. Then, rinsing with PBS (0.5 mL) and recollection was done. Next, the cells were incubated with SORT antibody (as control) and the next time to TXT@Fe_3_O_4_/PVA/Au-SORT nanoparticles (10 μg/mL in DMEM, 200 μL), at 4 °C for 1 h, and then rinsed for two times with PBS. Then, the cells were subjected to sheep anti-human-FITC (0.1 μg/mL) at 4 °C for 30 min, and again rinsed with PBS. Finally, the isotonic (0.9% w/v) saline solution was added and the sample was studied by flow cytometry.

#### Methyl tetrazolium assay

Three wells of a 96-well plate were considered for each condition, containing 200 μL of the samples. For the magnetic particles, dispersion was performed via ultrasonication (50 KHz, 100 W L^−1^) for 5 min, at 37 °C. HTB76 and NIH 3T3 cells (10^6^ CFU) were cultivated in DMEM (5.0 mL) and fetal bovine serum (FBS, 10%), at 37 °C and 95% humidity. After completion of the incubation times, the medium was removed and fresh DMEM (100 μL) plus 3-(4,5-dimethylthiazol-2-yl)−2,5-diphenyl tetrazolium bromide (MTT, 10%) were added to the wells. Then, the contents were incubated for additional 4 h, at the same conditions. Next, ca. 100 μL of DMSO was added to dissolve the crystals. Ultimately, the concentration of the formed crystals was estimated by an ELISA reader at 600 nm.

#### Scratch migration assay

The caov-4 (HTB76) cells were seeded on the 12-well plates at the density of 50 × 10^3^ cells/well. When the cells reached 90% of confluency, a scratch was made using a pipette tip. The cells were then rinsed with PBS (0.1 M, pH = 7.3) to remove the dyed cells. The solution of TXT in PBS, Ca(OH)_2_@Fe_3_O_4_/PVA/Au-SORT, and TXT@Fe_3_O_4_/PVA/Au-SORT (20 μg/mL) were placed in the wells and allowed to incubate for 24 h. Time-dependent bright-field images were taken after completion of the incubation process at 37 °C.

#### In vivo experiments

In all, 9–11 weeks old female mice were selected for the in vivo study. They received 2 × 10^6^ luciferase expressing HTB76 cells/mouse through subcutaneous injection in the right flank area. Totally, 21 days was programmed for pretreatment and treatment with 50 mg/kg/day in 100 μL saline of Ca(OH)_2_@Fe_3_O_4_/PVA/Au-SORT and TXT@Fe_3_O_4_/PVA/Au-SORT, respectively. The nine mice were divided into three groups; *G1* as control (only received saline), *G2* without any pretreatment, only received TXT@Fe_3_O_4_/PVA/Au-SORT on 8th, 10th, and 12th days, and *G3* pretreated for a week, then treated for 14 days. *G3* received Ca(OH)_2_@Fe_3_O_4_/PVA/Au-SORT on the first and third days, then received TXT@Fe_3_O_4_/PVA/Au-SORT, according to the *G2*’s program. The tail vein was used for the intravenous injection. After each time of injection, the mice were exposed to an external magnetic field (1 T) for 3 h, and then 9 h rest, afterward 15 min irradiation of near-infrared (NIR) (808 nm laser, 1.5 W/cm^2^, 5-mm spot diameter). To prepare the mice for bioluminescence imaging, intraperitoneal injection of D-luciferin (150 μg/kg) was carried out on the 20th day of the study. Finally, the mice were sacrificed after completion of the 21-day study via euthanization, and the grown tumors were excised and evaluated. We have complied with all relevant ethical regulations for animal testing and research. This study received protocol approval from Iran University of Science & Technology (IUST)—Chemistry Department.

#### Ex vivo tumor evaluation

Six tumor-bearing female mice were selected for a seven-day treatment, dividing into two groups; the first one including three mice as control just received blank saline, and the second one including three mice, intravenously received two dosages of Ca(OH)_2_@Fe_3_O_4_/PVA/Au-SORT (50 mg/kg/day in 100 μL saline), on first and third days of study. On the sixth day, DiR@Fe_3_O_4_/PVA/Au-SORT with the same dosage was injected into the mice of two groups. An external magnetic field (1 T) focusing on the right flank area was applied to the mice for 3 h, immediately post-injection. After 9 h, the NIR irradiation was performed for 15 min, focusing point on the local targeting. Finally, the mice were euthanized on the eighth day, and the organs and tumors were excised and evaluated.

#### H&E histopathological staining

A mixture of picric acid, formaldehyde (50 wt% in water), and glacial acetic acid with a volume ratio [12:4:1] was prepared and used for the fixture of the sliced organs, including liver, spleen, kidney, heart, lung, and grown tumor, with ca. 4-μm thickness. Then, staining was performed with a mixture of Hematoxylin and Eosin according to the procedures given in the literature^69^.

#### Phospho-H2AFX Staining

The slices of ovary tumor sections (4-μm thickness) were deparaffinized, hydrated, and rinsed (three times, for 5 min) with the deionized water. Then, the slices were fixed and blocked for an hour at room temperature, using goat serum (10% in PBS), and then washed with PBS (three times, for 5 min). The slices were incubated with rabbit anti-phospho-H2AFX at a 1:480 dilution in PBS with goat serum overnight at 4 °C. Afterward, the stained slices were rinsed (three times, for 5 min), then incubated in goat anti-rabbit Alexafluor 488 at a 1:400 dilution in PBS for an hour at room temperature and darkness. Ultimately, the slices were rinsed (three times, for 5 min) with PBS. Coverslip was placed with crystal mount containing 1.0 mg/mL propidium iodide with DAPI (Invitrogen).

#### Stability experiment on Fe_3_O_4_/PVA/Au carrier

The stability of Fe_3_O_4_/PVA/Au carrier was evaluated in three different media; phosphate buffer saline (PBS, 0.1 M, pH = 7.3), acetate buffer (AcB, 0.1 M, pH = 4.6), and human serum albumin (HAS, 20%). In each case, 50 mg of Fe_3_O_4_/PVA/Au particles were well dispersed in 25 mL of related medium through ultrasonication (50 KHz, 100 W L^−1^). Then, the samples were transferred into the glass cylindrical container and heated up to 37 ± 1 °C. Afterward, the circulatory system was set up using the pump and silicon vessels (clear vessel, with a diameter of 2.5 mm). Next, the cylindrical container was exposed to the green LED light (7 W) (in the case of LSPR condition), and the content was circulated for the following times; 12, 24, 36, 48, 60, and 72 h. After each time, the particles were collected at the end of the container using an external magnet (via holding that at the bottom of flask), washed, dried, and weighted with a high-precision digital balance. After completion of the process in 72 h, the particles were collected at the end of the container and a small portion of supernatant was withdrawn and sent to inductively coupled plasma analysis. The experiments were repeated three times, and the average values have been reported (*n* = 3).

#### Statistics and reproducibility

According to the rules, all practical sections have been performed on three repeated samples (at the same conditions) to guarantee the reproducibility of the results (*n* = 3). Then, STDEV and average values of the raw data were calculated by Excel. In figure captions, we defined the errors for each case study, as follows; STDEV represents the absolute errors, and %relative error was estimated via dividing the STDEV by the average value in each case, then multiplying by 100.

### Reporting summary

Further information on research design is available in the Nature Research Reporting Summary linked to this article.

## Supplementary information

Supplementary Information

Reporting Summary

## Data Availability

The raw data have been deposited in https://figshare.com/s/856399a0e33031621a7d. Also, some parts were reported in the SI section: the SI file includes information on the used reagents, solvents, tools, and equipment in this work. Also, the TEM and digital images of the synthesized gold nanoparticles have been illustrated in this section. Moreover, the flow cytometry density plots of MCF-7 cells have been given in the SI section. FTIR, EDX, PL, UV-DRS, DLS, and VSM analyses have been given in this section. Calculations of the ratios of different components in the presented drug delivery system based on CHN and EDX analyses, and drug content have been presented in the SI section (Supplementary Note 1). As well physiological stability and degradation experiments of the nanocarrier system have been described in this section. A graphical presentation of the work is illustrated as a supplementary figure.
