# Supplementary material for: A magnetic antibody-conjugated nano-system for selective delivery of Ca(OH)2 and taxotere in ovarian cancer cells
Source: Commun Biol. 2022 Sep 21;5:995. doi: 10.1038/s42003-022-03966-w (PMC9492675; doi:10.1038/s42003-022-03966-w)
Supplement: Supplementary file 1 — Supplementary Information [file 42003_2022_3966_MOESM1_ESM.pdf]

Supporting Information

A Magnetic Antibody-Conjugated Nano-System for Selective Delivery of Ca(OH)2 and Taxotere in Ovarian Cancer Cells

Reza Taheri-Ledari, Ehsan Zolfaghari, Simindokht Zarei-Shokat, Amir Kashtiaray, Ali Maleki**

Catalysts and Organic Synthesis Research Laboratory, Department of Chemistry, Iran University of Science and Technology, Tehran 16846-13114, Iran

**Corresponding authors E-mail addresses: r_taheri94@chem.iust.ac.ir; rezataheri13661206@gmail.com (R. Taheri-Ledari), maleki@iust.ac.ir (A. Maleki). Tel.: +98 21 77240640-50; fax: +98 21 73021584.*

Table of content

Content	Page
**Table S1**. The chemical used in this project.	**S2**
**Table S2**. The equipment used in this project.	**S3**
***FTIR spectroscopy*; Figure S1.** FTIR spectra the samples.	**S4**
***EDX spectroscopy*; Figure S2.** EDX spectra the samples.	**S5**
Supplementary Note 1.	**S6**
***PL and UV-DRS spectroscopy*; Figure S3**. PL emission and UV-DRS spectra of the samples.	**S7**
***VSM analysis;* Figure S4.** Room temperature VSM curves of the samples.	**S8**
**Figure S5**. TEM image of the synthesized AuNPs.	**S9**
**Figure S6**. Digital image of the synthesized AuNPs (colloidal solutions).	**S9**
***Zeta-sizer analysis*; Figure S7.** Intensity-based DLS curves of the samples.	**S10**
***Drug content and release;* Figure S8.** UV-vis spectra, and calibration curve of the standard TXT samples.	**S11**
***Stability and degradation;* Figure S9.** The obtained results from degradation experiment on drug carrier.	**S12**
**Table S3**. The obtained results from degradation experiment and ICP analysis.	**S13**
**Figure S10**. Flow cytometry FSC×SSC density plots of TXT@Fe_3O_4/PVA/Au-SORT nano-therapeutics over MCF-7 cells.	**S14**
**Supplementary figure**	**S15**
**Supplementary references**	**S16**

Table S1. The chemical used in this project.

Reagent	Brand & Purity
Solvents	Merck
Iron(II) chloride tetrahydrate	Sigma-Aldrich, ≥99.0%
Iron(III) chloride hexahydrate	Sigma-Aldrich, ≥99.0%
Tetraethyl orthosilicate	Sigma-Aldrich, 99.0%
Poly(vinyl alcohol), M_w= 72000	Sigma-Aldrich, ≥99.0%
(3-Chloropropyl) trimethoxysilane	Sigma-Aldrich, ≥97.0%
Calcium hydroxide	ACS reagent 95.0%
Taxotere	Sigma-Aldrich, Purum, ≥97.0%
Tetrachloroauric(III) acid trihydrate	Sigma-Aldrich, Supelco, 99%
Sodium borohydride	Sigma-Aldrich, 96.0%
Sodium citrate dihydrate	Sigma-Aldrich, ≥99.0%
MTT solution	Sigma-Aldrich, 98.0%
DMEM	Sigma-Aldrich
HSA (20%)	Octapharma (Vienna, Austria)
Crystal violet ($C_{25}N_3H_{30}Cl$)	Merck, ≥90.0%
Lugol's solution	Merck
3T3 and caov-4 cell lines*	NIH 3T3, Fibroblast National Research Center for Genetic Engineering and Biotechnology of Iran.<br>Caov- 4 (HTB-76), ATCC, Manassas, VA, USA
Sortilin 2D8-E3 mAb (SORT)	Avicenna Research Institute, Iran
DiR	Biotium (USA)
D-Luciferin	Sigma-Aldrich, ≥99.0%
Isoflurane	Merck
Paper filter	Whatman (grade 602h, Particle retention < 2µm)
Hematoxylin	Merck
Eosin	Sigma Aldrich (dye content ~99 %)

Expression of sortilin was knocked down using RNAi technology in the ovarian carcinoma cell line, Caov-4. Silencing of SORT1 expression was assessed using real-time qPCR and Western blot analyses. The living cells were cultured in their optimal conditions in RPMI-1640 (Gibco, Paisley, Scotland), containing 10% FBS (Gibco, Pais-ley, Scotland), 100 units/ml penicillin (ICN Biomedicals, Ohio) and 100 µg/ml streptomycin (Sigma, St. Louis, MO) at 37°C in a hu-midified incubator with 5% CO_2 atmosphere.

Table S2. The equipment used in this project.

**Instrument**	**Brand**
FTIR	Shimadzu FTIR-8400S
EDX	VEGA-TESCAN-XMU
FESEM	Hitachi S-5200
TEM	Philips CM200
VSM	Meghnatis Kavir Kashan Co., Kashan, Iran
DLS	Horiba (SZ-100)
ELISA	Bio-Tek ELx800
Flow cytometry	Agilent
UV-vis spectroscopy	Beckman DU640
Confocal microscopy	Zeiss LMS 700
Green LED light	SABA (7 W)
Thermometer	Fluke (572-2 infrared)
Ultrasound probe	Hielscher (UP100H)
Bioluminescence camera	PerkinElmer IVIS Spectrum
NIR irradiation	HSGD22-808AD1200
Ex vivo imaging	Maestro system (CRi, Inc., USA)
H&E Photographs	Inverted microscope (C2 plus system, Nikon, Japan)
Scratch images	Olympus (X53) microscope

FTIR spectroscopy

Briefly, in all three samples, the peak emerged at ca. 570 cm^{-1} comes from the Fe−O bond, and a sharp peak appeared at ca. 1088 cm^{-1} is attributed to the stretching vibrations of the Si−O−Si bands, which are abundantly found in the silica network.[1] Between the mentioned areas, there are some peaks that appeared at ca. 798 and 940 cm^{-1} coming from Si−OH and SiO−H, respectively.[2] These peaks confirm the presence of the –OH groups onto the surfaces. Another peak that is common in all samples, is a broad peak that emerged at ca. 3422 cm^{-1} attributing to the stretching vibrations of O−H bonds. Through a comparison between two spectra (a) an (b), it is found out that the peak intensity of the O−H bond is expectedly higher in the spectrum (b) confirming well combination of the PVA matrix that contains numerous –OH groups in its chemical structure.[3] In the spectra (b) and (c) that are related to Fe_3O_4@SiO_2-PVA and TXT@Fe_3O_4/PVA/Au-SORT particles, respectively, a small peak appeared at ca. 2924 cm^{-1} that is related to the vibrations of C─H bonds with hybridation sp3, present in the structure of the PVA, chloropropyl silane (CPS), and TXT, and SORT antibody. Two broad peaks have been appeared at ca. 2355 and 2430 in the spectrum (b), which may origin from the adsorbed CO_2 by the jelly PVA layer, and also some impurities in KBr tablet during sample preparation. Overall, successful incorporation of the TXT in the structure and surface conjugation of SORT can be corroborated by the appearance of several peaks that appeared at ca. 1400-1800 cm^{-1}, which coming from the C–N, C=C, and C=O groups in the structure of SORT and TXT (spectrum c).[4]

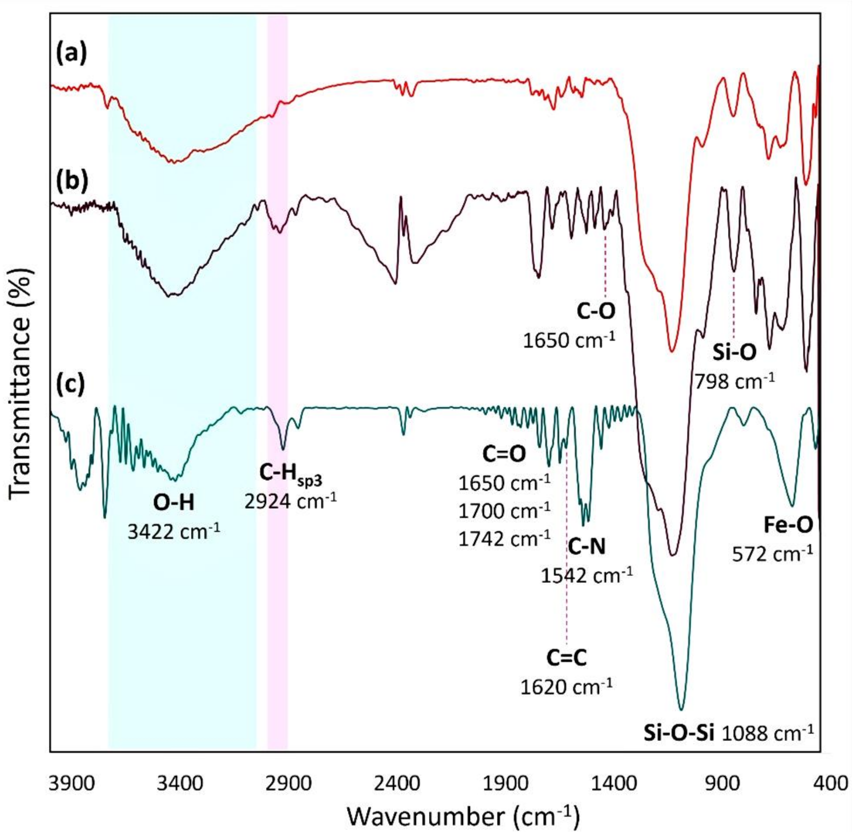

Figure S1. FTIR spectra of Fe_3O_4@SiO_2 (a), Fe_3O_4@SiO_2-PVA (b), and TXT@Fe_3O_4/PVA/Au-SORT (c) particles.

EDX spectroscopy

In order to highlight the structural differences between Fe_3O_4@SiO_2, Fe_3O_4@SiO_2-PVA, Fe_3O_4@PVA-CPS, and TXT@Fe_3O_4/PVA/Au-SORT samples, energy-dispersive X-ray (EDX) spectrometer was used as an elemental analysis (Fig. 3). As shown in spectra (a) and (b), a characteristic peak for carbon element was appeared at 0.285 keV after surface coating of the Fe_3O_4@SiO_2 particles by PVA. Also, the peak intensity of silicon atom decreased after this stage, meaning that a new layer has coated the silica network. In spectrum (c) that is related to Fe_3O_4@PVA-CPS, the peak intensity of the carbon element was increased after modification of the surfaces by CPS (propyl groups). In the same spectrum, as a confirmation for the successful formation of the covalent bonds between the CPS and outer layer of the PVA network, the presence of the chlorine atom is considered by a peak appeared at 2.655 keV. In the spectrum of TXT@Fe_3O_4/PVA/Au-SORT (spectrum d), the presence of the AuNPs is confirmed by the appeared peaks at ca. 8.6 and 9.8 keV. Moreover, separation of the chlorine elements due to the covalent attachment of the SORT antibodies has been verified by disappearance of the chlorine's peak. In fact, execution of a nucleophilic substitution reaction by the amine groups (in the lysine amino acids of SORT) on the C–Cl bonds (in Fe_3O_4@PVA-CPS particles) is the most probable reaction at this stage. In the same spectrum, the appearance of a new peak at ca. 2.6 keV related to the sulfur atoms well verifies the present of the SORT antibodies onto the surfaces. Based on the CHN and EDX data, *TXT/Fe* and *Au/Fe* ratios in the structure of TXT@Fe_3O_4/PVA/Au-SORT have also been estimated to be ca. 0.05 and 0.0065, respectively. Related calculation methods have been presented in the Supporting Information (SI) file in the electronic version of this article.

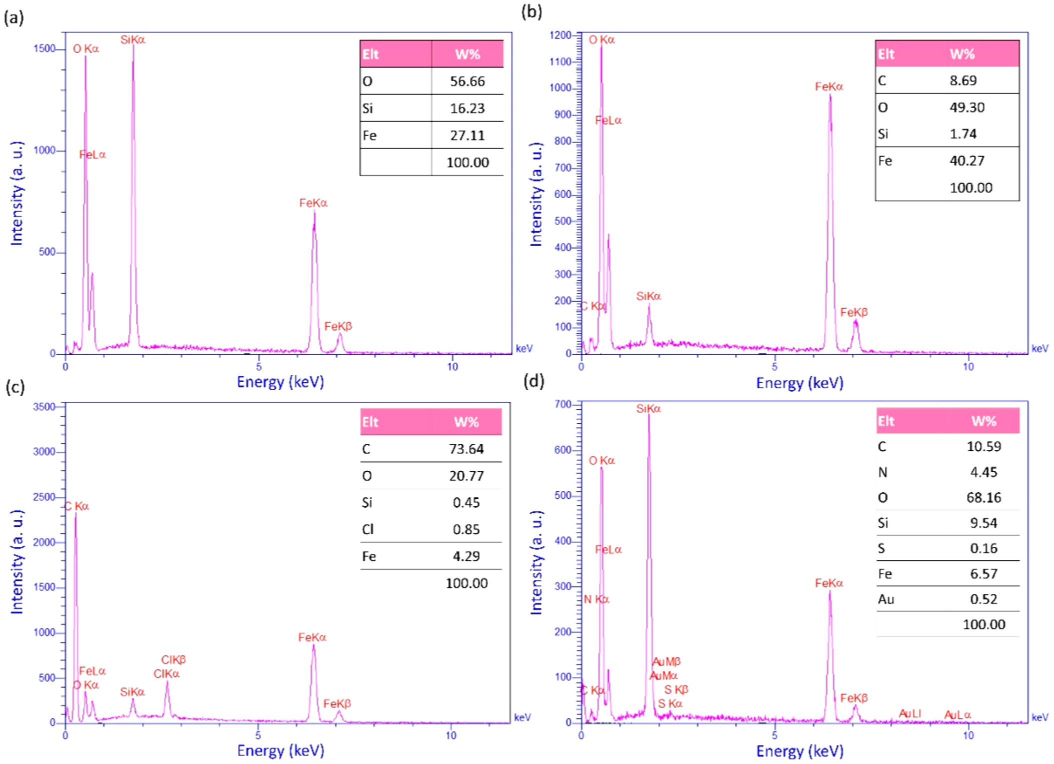

Figure S2. EDX spectra and quantitative results of Fe_3O_4@SiO_2 (a), Fe_3O_4@SiO_2-PVA (b), Fe_3O_4@PVA-CPS (c), and TXT@Fe_3O_4/PVA/Au-SORT (d) particles.

Supplementary Note 1.

Calculation of the ratios of different components in TXT@Fe_3O_4/PVA/Au-SORT based on CHN and EDX analyses

A) TXT

TXT amounts can be estimated from the CHN analysis based on the carbon difference between products **4** and **5** (see Figure 1 in the main text and a schematic drawing below).

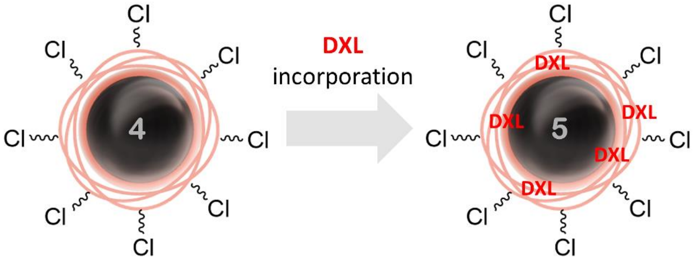

% C	% H	% N
14.52	1.88	0.11

% C	% H	% N
25.21	2.08	0.13

$Carbon\ content\ corresponding\ to\ the\ encapsulated\ TXT\ is\ then: 25.21 - 14.52 = 10.69\ wt\%$

Considering the chemical formula and molecular weight of TXT ($C_{43}H_{53}NO_{14}$, 807.9 g/mol) and the carbon quota in the formula (43 × 12 amu of C = 516 g/mol), the weight percentage (wt%) of the encapsulated TXT (**X**) is: 10.7 × 807.9 / 516.0 = 16.7 wt% (proportion ratio is as below)

TXT: 807.9 g/mol → 516.0 g/mol (belongs to carbon in TXT)
Encapsulated TXT: **X** → 10.7 (carbon quota in the encapsulated TXT in product **5**)

So, the moles of the encapsulated TXT per total mass is: 16.7/807.9 = 0.02 mol

B) TXT/Fe ratio in product 6

From the EDX analysis, 22.8 wt% belongs to Fe. Considering the molecular weight of the Fe (55.8 g/mol), the moles of the Fe per total mass is: 22.8/55.8 = 0.4

So, the TXT/Fe molar ratio is: 0.02/0.4 = **0.05**

C) Au/Fe ratio in product 6

From the EDX analysis, 0.52 wt% belongs to Au. Considering the molecular weight of the Au (197.0 g/mol), the moles of the Au per total mass is: 0.52/197.0 = 0.0026

So, the Au/Fe molar ratio is: 0.0026/0.4 = **0.0065**

PL and UV-DRS spectroscopy

To perform more characterization on complexation and incorporation of TXT into the Fe_3O_4/PVA-CPS system, photoluminescence (PL) emission and ultraviolet-visible differential reflectance spectroscopy (UV-DRS) spectra of TXT@Fe_3O_4@PVA-CPS were investigated and compared with the individual TXT and Fe_3O_4/PVA-CPS as the controls. As presented in Fig. 4a, the PL activity of the TXT@Fe_3O_4@PVA-CPS particles (red-plotted curve) coincides with the individual TXT (yellow-plotted curve) at ca. 110 nm, confirming that TXT has been successfully loaded into the Fe_3O_4/PVA-CPS carrier system.[5] The PL peak intensity for the TXT@Fe_3O_4@PVA-CPS is less than the individual TXT because a small percentage of the total weight of TXT@Fe_3O_4@PVA-CPS in formed by TXT. In Fig. 4b, the UV-Vis reflectance activity of the samples were compared with each other. As is observed in the obtained spectra, the complexation of the TXT on the Fe_3O_4/PVA-CPS system is verified by the peak appeared at ca. 230 nm.[6] The rest of the reflectance activity in a range of 235-440 nm in the curves related to Fe_3O_4/PVA-CPS (green-plotted) and TXT@Fe_3O_4@PVA-CPS (red-plotted) is attributed to the Fe_3O_4 and PVA components.[7] Based on the obtained results from PL and UV-DRS analyses, the incorporation of the TXT in the Fe_3O_4/PVA-CPS system through the mentioned physico-chemical interactions can be corroborated.

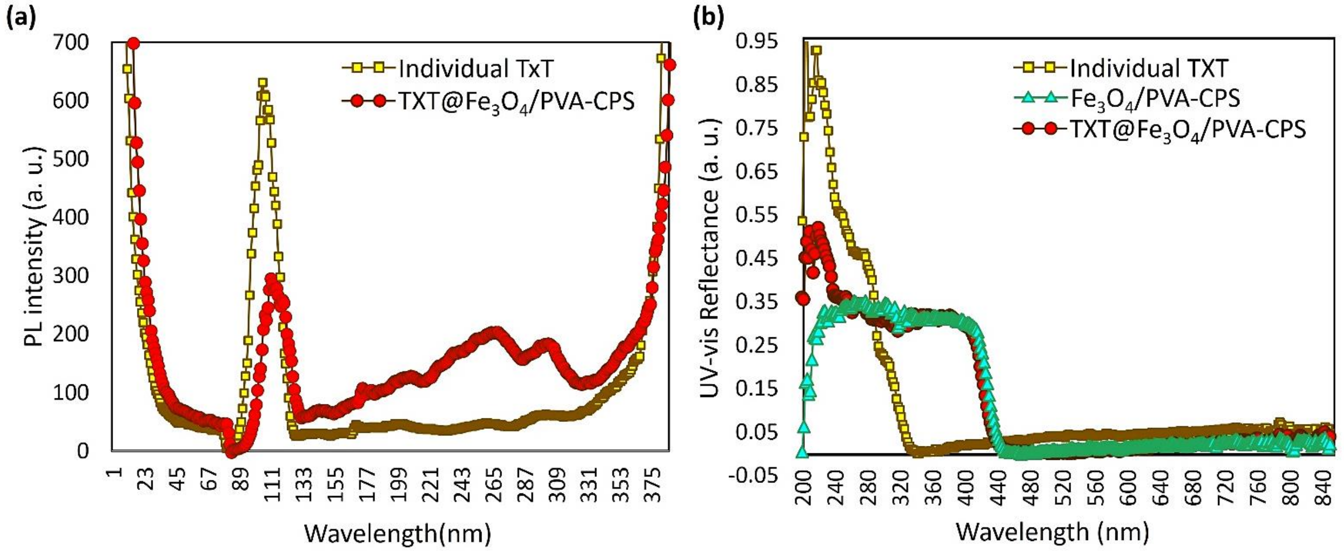

Figure S3. (a) PL emission and (b) UV-DRS spectra of TXT@Fe_3O_4@PVA-CPS cargoe, individual TXT, and Fe_3O_4/PVA-CPS (as control).

VSM analysis

Magnetic behavior of $Fe_3O_4@SiO_2$, $Fe_3O_4@SiO_2$-PVA and TXT@Fe_3O_4/PVA/Au-SORT was investigated by a vibrating-sample magnetometer (VSM) instrument to find out how much magnetism is diminished during the composition processes. As exhibited in Fig. 5b, a significant magnetic property has been observed for $Fe_3O_4@SiO_2$ (curve 1) since the magnetic saturation has been occurred in ca. 47 emu/g through applying an external magnetic field with the power of 7K Oe. The magnetic-hysteresis curves of $Fe_3O_4@SiO_2$-PVA and TXT@Fe_3O_4/PVA/Au-SORT (curves 2 and 3, respectively) revealed that the magnetic property is decreased to ca. 15 emu/g by coating the $Fe_3O_4@SiO_2$ core with the PVA polymeric shell. Obviously, the magnetism is generally decreased by combing the non-magnetic agents in the composites.[12] Although the magnetic property has been decreased after the addition of the more layers to the composite, this amount of the magnetism experimentally works for the convenient separation/conduction of the particles during the preparation/treatment processes. As another important point in the VSM curves, an additional partial reduction (ca. 6 emu/g) after incorporation of the TXT and AuNPs and surface functionalization with SORT antibodies should be noticed. This observed reduction in the magnetic behavior can be another confirmation on the successful combination of further non-magnetic materials.

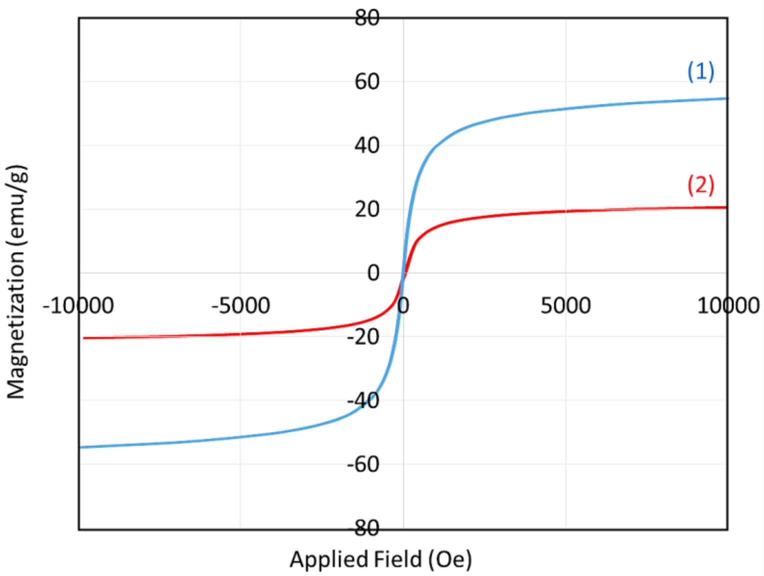

Figure S4. Room temperature VSM curves of the $Fe_3O_4@SiO_2$ (1) and TXT@Fe_3O_4/PVA/Au-SORT (2).

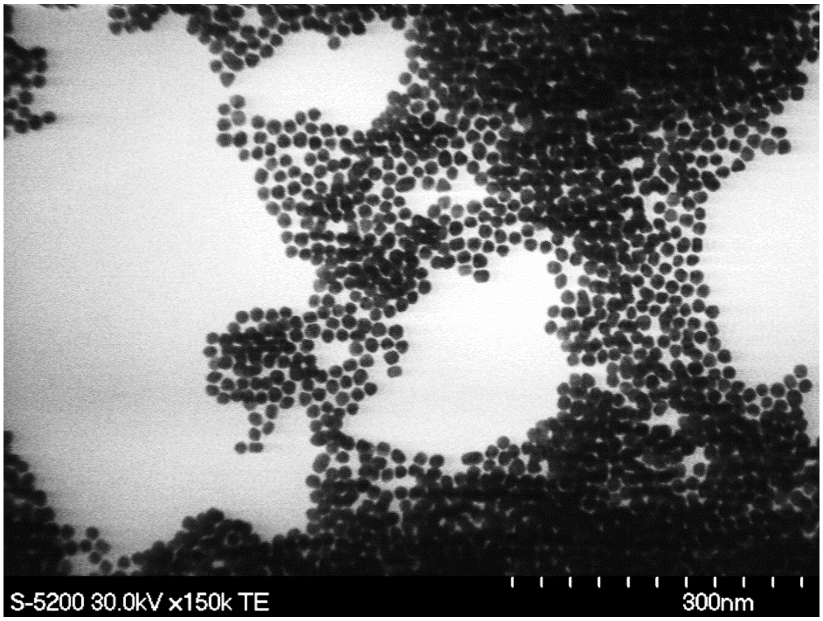

Figure S5. TEM image of the synthesized AuNPs.

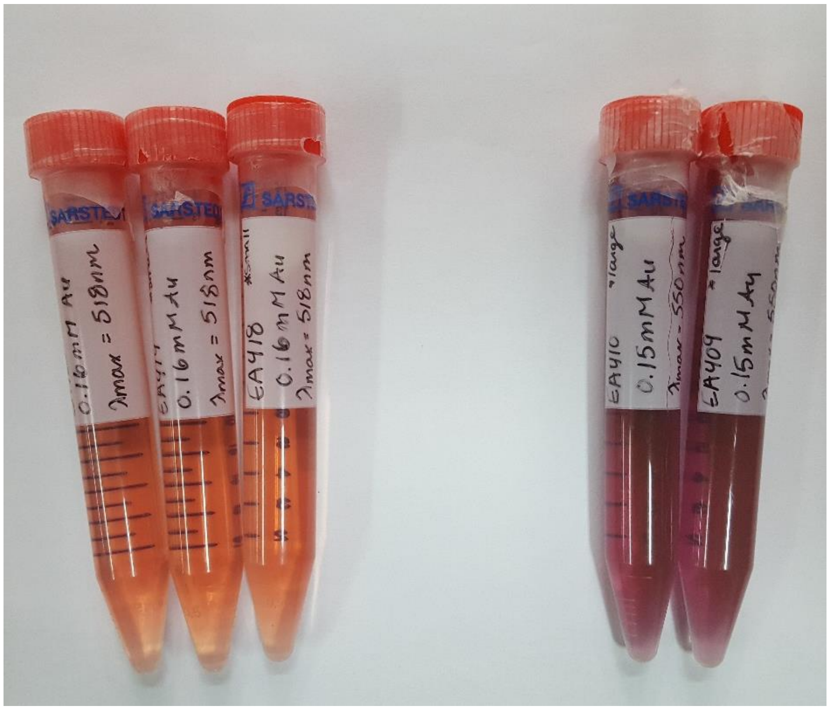

Figure S6. Digital image of the synthesized AuNPs (colloidal solutions).

Zeta-sizer analysis

As shown in Fig. 7, a sharp and relatively slim peak with a small polydispersity index (PDI) (ca. 1.2) is observed for the dilute sample. Therefore, it can be deduced that well dispersion of the particles is obtained in the concentration of 10 μg/mL. From this peak, the mean size of the particles in ca. 75 nm is confirmed that is in great correspondence with the EM imaging results (Fig. 6). To screen how particle aggregation depends on the concentration, a few more concentrated samples (20, 30, and 50 μg/mL) were prepared and experimented. This matter may be considered as one of the limiting factors for drug dosage administration. As is observed, particles aggregation rises in the samples proportional to increasing the concentration. In fact, the observed rise in the average size value of the particles' origins from the particles aggregation, which is enhanced by increasing the concentration. Based on the particles' dispersion/aggregation states, it is deduced that the most appropriate dosage of the TXT@Fe_3O_4/PVA/Au-SORT is ca. 50 μg/mL, since in the case of 50 μg/mL a separated broad peak with a large PDI has been observed showing high grades of the aggregation. This aggregation causes the effective wavelength in the LSPR heating to shift to the higher values closer to NIR irradiation. It seems that in the case of the aggregated samples with a large value of the DPI, the equipment could not give a single peak showing the average size of the particles.[4,11] That is why there are two distinct peaks in the case of the sample with 50 μg/mL concentration. However, as the main contributors to particles aggregation, jelly nature and magnetism of the particles can be mentioned, which lead them to attract each other in the colloidal phase.[4,11]

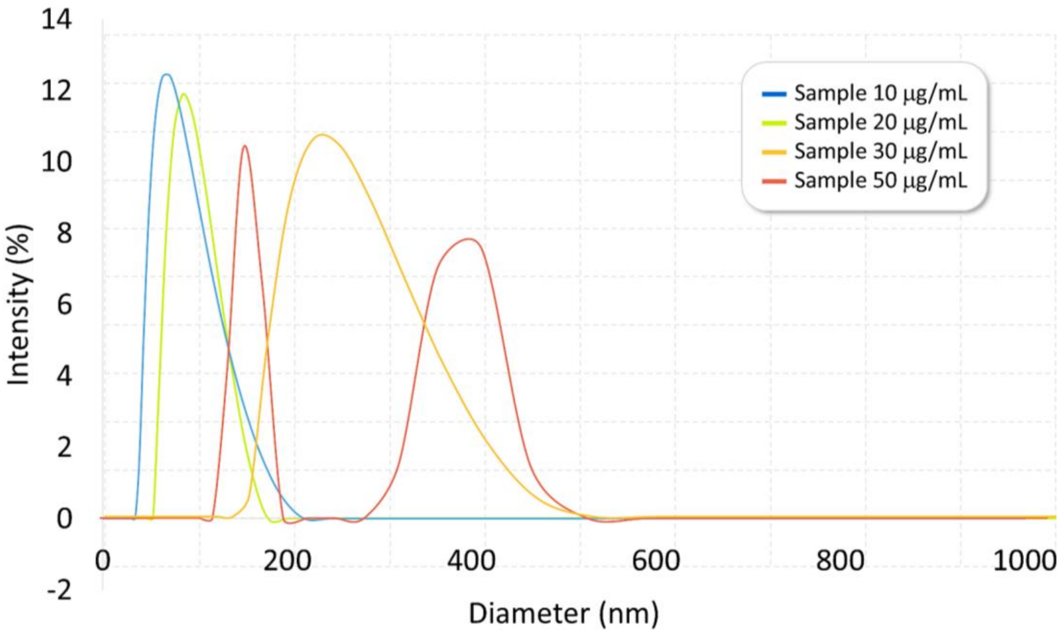

Figure S7. Intensity-based DLS measurements of particle size (zeta-average) and size distribution of TXT@Fe_3O_4/PVA/Au-SORT, in different concentrations.

Estimation of TXT content in the composite structure of TXT@Fe_3O_4/PVA/Au-SORT

To investigate how much TXT is incorporated in the composite, UV-vis spectroscopy was used.[13] For this purpose, a calibration curve was initially obtained from UV-vis absorption activity of five standard solutions of TXT in PBS (0.1, pH = 7.3) with the concentrations of 5, 10, 15, 25, and 30 ppm, at λ_{max} = 230 nm (Figure S8a in the SI section).[4,11] Then, the related line equation based on the concentration (ppm)-absorption (a. u.) was obtained, which confirmed high precision in the preparation of the standard solutions (R^2 = 0.997) (Figure S8b). Afterward, the particles of TXT@Fe_3O_4/PVA/Au-SORT were well grinded via ball-milling (25 Hz) and dispersed in DMSO via ultrasonication (50 KHz, 100 W L^{-1}), at 40 °C for 1 h. Then, the mixture was vigorously stirred for an additional hour at the same temperature. Next, the particles were magnetically separated and the rest was filtrated by paper filters to obtain a clear solution. The obtained solution was diluted (2 mL to 25 mL) with PBS and studied by UV-vis spectroscopy. According to the detailed calculation method (presented below), the loaded TXT in the nano-carriers was estimated ca. 13.3 ±1.9 wt%.

$$\text{TXT@Fe3O4/PVA/Au} - \text{SORT}\ (50\ mg)\ in\ DMSO \xrightarrow[2)\ Stir, 40\ deg., 2\ h]{1)\ Ultrasonication} colloidal\ mixture\ \left(2.0\ \frac{mg}{mL}\right) \xrightarrow[2)\ Filtration]{1)\ Magnetic\ separation} clear\ solution \xrightarrow{Dilution\ (2.0\ ml\ to\ 25\ mL)} \xrightarrow{UV-vis\ apsorption} A = 0.492\ (a.u.) \quad \textbf{(Eq. 1)}$$

$$\boldsymbol{Line\ equation}: \boldsymbol{X\ (ppm)} = \frac{\boldsymbol{y-0.0335}}{\boldsymbol{0.0214}} \xrightarrow{(y=0.492)} X = 21.4\ ppm \xrightarrow{(\times\ ^{25}/_{2})} 267.5\ ppm = 0.267\ \frac{mg}{mL} \xrightarrow{(in\ 25\ mL\ DMSO)} 0.267 \times 25 = 6.675\ (\text{mg of TXT in 25 mL DMSO}) \quad \textbf{(Eq. 2)}$$

$$\frac{6.675\ mg\ (Released\ DXL\ from\ 50\ mg\ of\ \text{TXT@Fe3O4/PVA/Au–SORT})}{50\ mg\ (Weight\ of\ dispersed\ particles\ in\ DMSO)} \times 100 = 13.35\ wt\%\ (TXT\ in\ \text{TXT@Fe3O4/PVA/Au} - \text{SORT}) \quad \textbf{(Eq. 3)}$$

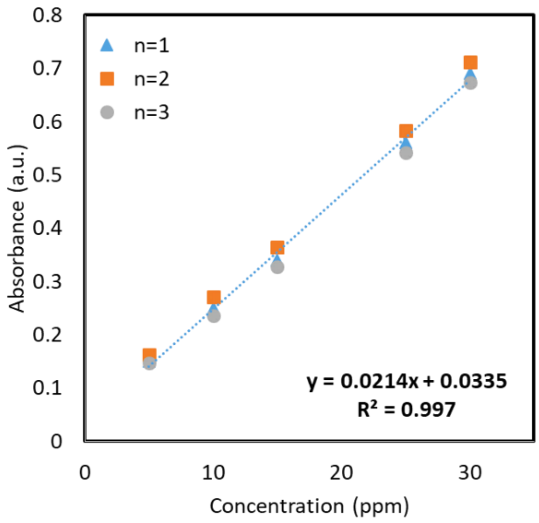

Figure S8. Calibration curve of the standard TXT samples in PBS. The stock solution of TXT was prepared in ethanol with a concentration of 500 ppm, then the samples were diluted with PBS (0.1 M, pH = 7.3). The limit of detection (LOD) and limit of quantification (LOQ) values for the calibration curve were calculated 1.31 and 3.97 ppm, respectively (n: repetition time).

Screening of physiological stability and degradation states of Fe_3O_4/PVA/Au nanocarrier

To investigate the stability of the Fe_3O_4/PVA/Au composite particles as the main skeleton of the designed TXT@Fe_3O_4/PVA/Au-SORT nano-cargo, different conditions and media were experimented in a simulated circulatory system including a bilateral pump, cylindrical glass container, and silicon vessels. To have a true comparison of the effect of different media, PBS (0.1 M, pH = 7.3) and AcB (0.1 M, pH = 4.6) were used in the presence and absence of the LSPR conditions (by LED, 7 W). Moreover, human serum albumin (HSA) was used to better realize the effect of the biological environment. Practically, three dispersions of Fe_3O_4/PVA/Au particles in PBS, AcB, and HSA 20% (with the concentration of 2.0 mg/mL, at 37 °C) were prepared in the cylindrical container and exposed to the green LED light. The prepared mixtures were circulated for 12, 24, 36, 48, 60 and 72 hours, then the particles were magnetically separated after completion of the process, washed, dried and weighted with a high-precision digital balance. As well, the supernatants were analyzed by inductively coupled plasma (ICP) analysis to evaluate the released ions from degraded particles after 72 hours. As presented in Fig. 16 and Table 2, the highest value of degradation has been observed in AcB medium (pH = 4.6) confirming well stability of the Fe_3O_4/PVA/Au nanocarrier in the physiological environment, and quick digestion in acidic environment inside the cancer cells.[14] Based on the obtained results, the half-life value for the Fe_3O_4/PVA/Au nanocarrier at normal and acidic conations are estimated to be ca. 96 and 58 hours, respectively.

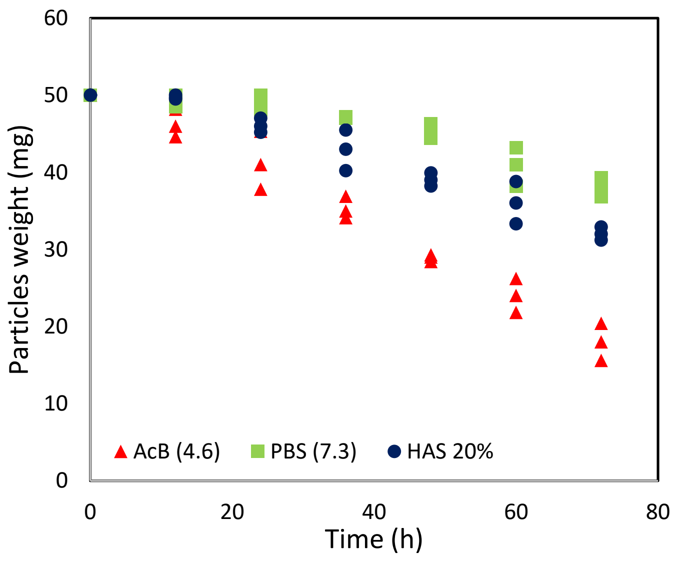

Figure S9. The obtained results from degradation experiment on the Fe_3O_4/PVA/Au nanocarrier during a 72-hour process.

Table S3. The obtained results from degradation experiment and ICP analysis related to Fe_3O_4/PVA/Au nanocarrier during a 72-hour process.

Medium	Concentration of Fe (ppm)*	Concentration of Au (ppm)*	Weight loss (mg) ±STDEV
AcB (0.1 M, pH = 4.6)	61.23	4.47	32.3 ±5.5
PBS (0.1 M, pH = 7.3)	19.58	0.95	12.0 ±5.0
HAS (20%)	28.17	1.85	18.2 ±5.5

** The ICP analysis was carried out on the samples of supernatants of three different conditions; The temperature of the mixtures was maintained at (37 ±1.0) °C; The LSPR condition was applied through 10 min irradiation of a green LED light (7 W) per each 30-minute circulation.*

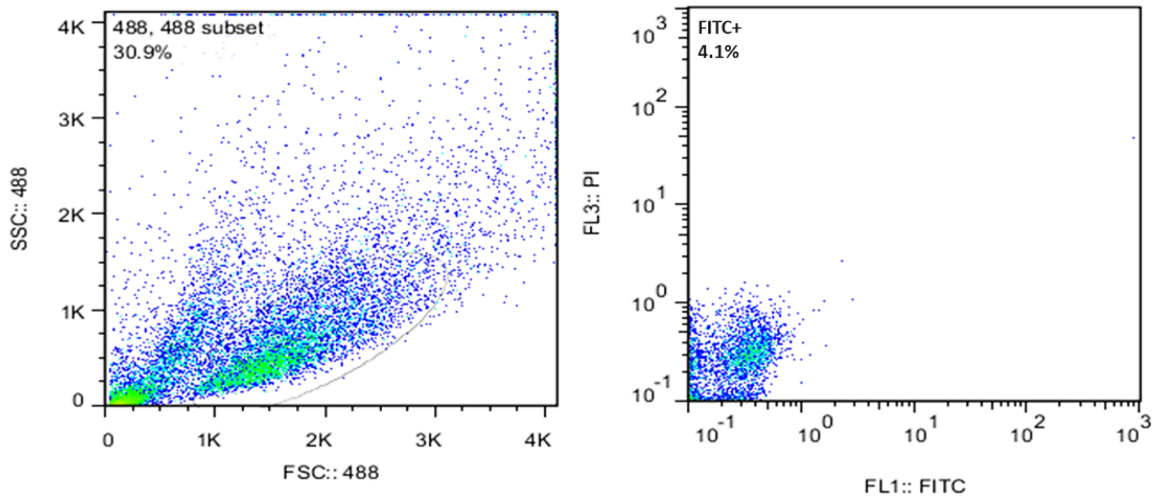

Figure S10. Flow cytometry FSC×SSC density plots of TXT@Fe_3O_4/PVA/Au-SORT nano-therapeutics over MCF-7 cells, ungated (left panel) and FITC-gated (right panel).

Supplementary figure

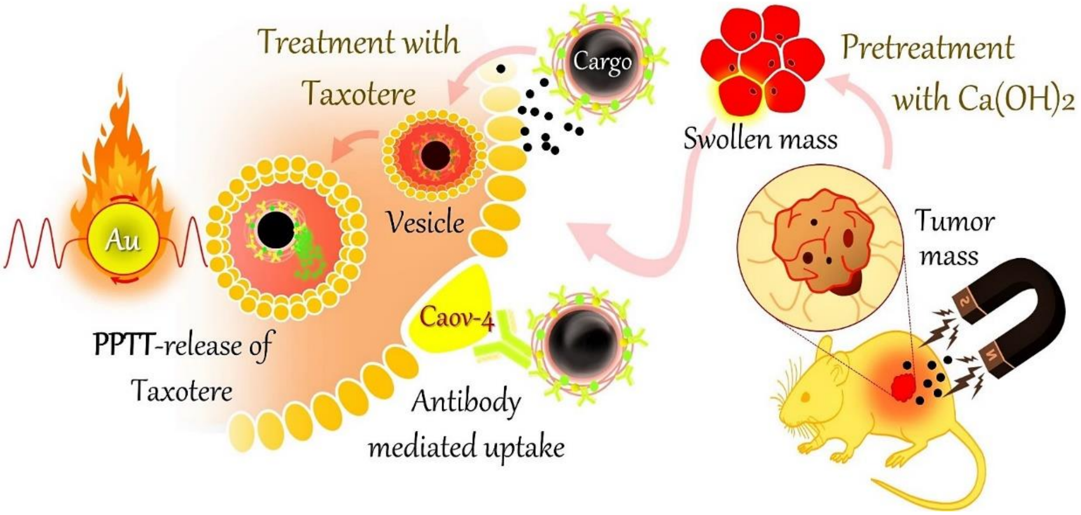

Supplementary references

1 A. Maleki, R. Taheri-Ledari, J. Rahimi, M. Soroushnejad, Z. Hajizadeh, Effective Interplay between Isothiazolone Rings and Silanol Groups at Silver/Iron Oxide Nanocomposite Surfaces. *ACS Omega*, 4, 10629-10639 (2019).

2 R. Taheri-Ledari, J. Rahimi, A. Maleki, Method Screening for Conjugation of the Small Molecules onto the Vinyl-coated Fe_3O_4/Silica Nanoparticles: Highlighting the Efficiency of Ultrasonication,.*Mater. Res. Express*, 7, 015067 (2020).

3 V. Soltaninejad, M. R. Ahghari, R. Taheri-Ledari, A. Maleki, Bifunctional PVA/ZnO/AgI/Chlorophyll Nanocomposite Film: Enhanced Photocatalytic Activity for Degradation of Pollutants and Antimicrobial Property under Visible-Light Irradiation. *Langmuir*, 37, 4700-4713 (2021).

4 R. Taheri-Ledari, W. Zhang, M. Radmanesh, S. S. Mirmohammadi, A. Maleki, N. Cathcart, V. Kitaev, Multi-Stimuli Nanocomposite Therapeutic: Docetaxel Targeted Delivery and Synergies in Treatment of Human breast Cancer Tumor. *Small*, 16, 2002733 (2020).

5 J. Liu, R. Li, and B. Yang, Carbon Dots: A New Type of Carbon-Based Nanomaterial with Wide Applications. *ACS Cent. Sci*. 6, 2179–2195 (2020).

6 H. Cheng, H. Liu, Y. Zhang, G. Zou, Interaction of the docetaxel with human serum albumin using optical spectroscopy methods. *J. Lumin*. 129, 1196-1203 (2009).

7 J. Yang, L. Fan, Y. Xu, J. Xia, Iron oxide nanoparticles with different polymer coatings for photothermal therapy. *J. Nanoparticle Res.* 19, 333 (2017).

8 J. Li, J. Wang, J. Li, X. Yang, J. Wan, C. Zheng, Q. Du, G. Zhou, X. Yang, Fabrication of Fe_3O_4@ PVA Microspheres by One-Step Electrospray for Magnetic Resonance Imaging during Transcatheter Arterial Embolization. *Acta Biomater.* 131, 532-543 (2021).

9 A. Maleki, M. Niksefat, J. Rahimi, R. Taheri-Ledari, Multicomponent Synthesis of pyrano [2, 3-d] pyrimidine Derivatives Via a Direct One-Pot Dtrategy eExecuted by Novel Designed Copperated Fe_3O_4@ Polyvinyl alcohol Magnetic Nanoparticles. *Mater. Today Chem.* 13, 110-120 (2019).

10 J. Rahimi, R. Taheri-Ledari, M. Niksefat, A. Maleki, Enhanced Reduction of Nitrobenzene Derivatives: Effective Strategy Executed by Fe_3O_4/PVA-10% Ag as a Versatile Hybrid Nanocatalyst. *Catal. Commun.* 134, 105850 (2020).

11 R. Taheri-Ledari, W. Zhang, M. Radmanesh, N. Cathcart, A. Maleki, V. Kitaev, Plasmonic Photothermal Release of Docetaxel by Gold Nanoparticles Incorporated onto Halloysite Nanotubes with Conjugated 2D8-E3 Antibodies for Selective Cancer Therapy. *J. Nanobiotech.* 19, 1-21 (2021).

12 R. Taheri-Ledari, A. Maleki, *Magnetic Nanoparticle-Based Hybrid Materials*, 619-636 (Woodhead Publ. Ser., 2021)

13 S. Thambiraj, R. Vijayalakshmi, D. R. Shankaran, An Effective Strategy for Development of Docetaxel Encapsulated Gold Nanoformulations for Treatment of Prostate Cancer. *Sci. Rep.* 11, 1-17 (2021).

14 Y. Kato, S. Ozawa, C. Miyamoto, Y. Maehata, A. Suzuki, T. Maeda, Y. Baba, Acidic extracellular microenvironment and cancer. *Cancer Cell Int*. 13, 89 (2013).
