# Supplementary material for: A magnetic antibody-conjugated nano-system for selective delivery of Ca(OH)2 and taxotere in ovarian cancer cells
Source: Commun Biol. 2022 Sep 21;5:995. doi: 10.1038/s42003-022-03966-w (PMC9492675; doi:10.1038/s42003-022-03966-w)
Supplement: Supplementary file 2 — Reporting Summary [file 42003_2022_3966_MOESM2_ESM.pdf]

nature portfolio

Corresponding author(s): COMMSBIO-22-0639-T

Last updated by author(s): Jun 21, 2022

Reporting Summary

Nature Portfolio wishes to improve the reproducibility of the work that we publish. This form provides structure for consistency and transparency in reporting. For further information on Nature Portfolio policies, see our Editorial Policies and the Editorial Policy Checklist.

Statistics

For all statistical analyses, confirm that the following items are present in the figure legend, table legend, main text, or Methods section.

n/a	Confirmed	
☐	☒	The exact sample size (n) for each experimental group/condition, given as a discrete number and unit of measurement
☐	☒	A statement on whether measurements were taken from distinct samples or whether the same sample was measured repeatedly
☐	☒	The statistical test(s) used AND whether they are one- or two-sided<br>*Only common tests should be described solely by name; describe more complex techniques in the Methods section.*
☐	☒	A description of all covariates tested
☐	☒	A description of any assumptions or corrections, such as tests of normality and adjustment for multiple comparisons
☐	☒	A full description of the statistical parameters including central tendency (e.g. means) or other basic estimates (e.g. regression coefficient) AND variation (e.g. standard deviation) or associated estimates of uncertainty (e.g. confidence intervals)
☐	☒	For null hypothesis testing, the test statistic (e.g. F, t, r) with confidence intervals, effect sizes, degrees of freedom and P value noted<br>*Give P values as exact values whenever suitable.*
☐	☒	For Bayesian analysis, information on the choice of priors and Markov chain Monte Carlo settings
☐	☒	For hierarchical and complex designs, identification of the appropriate level for tests and full reporting of outcomes
☐	☒	Estimates of effect sizes (e.g. Cohen's d, Pearson's r), indicating how they were calculated

Our web collection on statistics for biologists contains articles on many of the points above.

Software and code

Policy information about availability of computer code

Data collection	No special software was used,
Data analysis	No special software was used,

For manuscripts utilizing custom algorithms or software that are central to the research but not yet described in published literature, software must be made available to editors and reviewers. We strongly encourage code deposition in a community repository (e.g. GitHub). See the Nature Portfolio guidelines for submitting code & software for further information.

Data

Policy information about availability of data

All manuscripts must include a data availability statement. This statement should provide the following information, where applicable:

- Accession codes, unique identifiers, or web links for publicly available datasets
- A description of any restrictions on data availability
- For clinical datasets or third party data, please ensure that the statement adheres to our policy

The raw data has been deposited in https://figshare.com/s/856399a0e33031621a7d. Also, some parts reported in the SI section.

Field-specific reporting

Please select the one below that is the best fit for your research. If you are not sure, read the appropriate sections before making your selection.

☒ Life sciences ☐ Behavioural & social sciences ☐ Ecological, evolutionary & environmental sciences

For a reference copy of the document with all sections, see nature.com/documents/nr-reporting-summary-flat.pdf

Life sciences study design

All studies must disclose on these points even when the disclosure is negative.

Sample size	The sample size was estimated by electron microscopy and dynamic-light scattering methods, and the statistical data were recorded by the used equipment.
Data exclusions	No data was excluded from the analyses.
Replication	All samples replication were successful.
Randomization	Randomization is not relevant to our study, as no clinical test has been performed on the samples.
Blinding	Blinding was not peformed because it was not needed at all.

Reporting for specific materials, systems and methods

We require information from authors about some types of materials, experimental systems and methods used in many studies. Here, indicate whether each material, system or method listed is relevant to your study. If you are not sure if a list item applies to your research, read the appropriate section before selecting a response.

Materials & experimental systems

n/a	Involved in the study	
☐	☒	Antibodies
☐	☒	Eukaryotic cell lines
☒	☐	Palaeontology and archaeology
☐	☒	Animals and other organisms
☒	☐	Human research participants
☒	☐	Clinical data
☒	☐	Dual use research of concern

Methods

n/a	Involved in the study	
☒	☐	ChIP-seq
☐	☒	Flow cytometry
☒	☐	MRI-based neuroimaging

Antibodies

Antibodies used	Sortilin 2D8-E3 mAb (SORT)
Validation	https://www.ncbi.nlm.nih.gov/pmc/articles/PMC4147104/

Eukaryotic cell lines

Policy information about cell lines

Cell line source(s)	HTB76 and 3T3 cells; ATCC
Authentication	Non cell lines used in this work were authenticated.
Mycoplasma contamination	The cell lines were not tested for micoplasma contamination.
Commonly misidentified lines (See ICLAC register)	There is no misidentified cell line in this work.

Animals and other organisms

Policy information about studies involving animals; ARRIVE guidelines recommended for reporting animal research

Laboratory animals	9-11 weeks old female mice were used.

Wild animals	The study did not involved the wild animals.
Field-collected samples	The study did not involved samples collected from the field.
Ethics oversight	No ethical approval was needed for this work because all procedures were carried out according to the literature and previously approved reports.

Note that full information on the approval of the study protocol must also be provided in the manuscript.

Flow Cytometry

Plots

Confirm that:

☒ The axis labels state the marker and fluorochrome used (e.g. CD4-FITC).

☒ The axis scales are clearly visible. Include numbers along axes only for bottom left plot of group (a 'group' is an analysis of identical markers).

☒ All plots are contour plots with outliers or pseudocolor plots.

☒ A numerical value for number of cells or percentage (with statistics) is provided.

Methodology

Sample preparation	In a sterilized glass tube (13 by 100 mm), HTB76 cells (106 CFU) were stained with trypan blue (1% v/v, four drops) in PBS (0.1 M, pH = 6.8), diluted with DMEM and Lugol's solution. Next, the cells were rinsed with PBS and recollected via centrifugation (2K rpm, 5 min). Then, cell counting was carried out via excitation at 488 nm. Afterward, rinsing with antibody (10 □g/mL) was performed in sheep's serum (5 wt%) at 4 °C for 30 min, to clock unspecific sites. Then, rinsing with PBS (0.5 mL) and recollection was done. Next, the cells were incubated with SORT antibody (as control) and the next time to TXT@Fe3O4/PVA/Au-SORT nanoparticles (10 □g/mL in DMEM, 200 □L), at 4 °C for 1 h, and then rinsed for two times with PBS. Then, the cells were subjected to sheep anti-human-FITC (0.1 □g/mL) at 4 °C for 30 min, and again rinsed with PBS. Finally, the isotonic (0.9% w/v) saline solution was added and the sample was studied by flow cytometry.
Instrument	Agilent
Software	Nor available; This analysis was performed by other centers.
Cell population abundance	*Describe the abundance of the relevant cell populations within post-sort fractions, providing details on the purity of the samples and how it was determined.*
Gating strategy	FITC-gating was performed according to the procedure at https://www.stemcell.com/considerations-for-facs-gating.html

☒ Tick this box to confirm that a figure exemplifying the gating strategy is provided in the Supplementary Information.
